# Associations of perceived neighbourhood and home environments with sedentary behaviour among adolescents in 14 countries: the IPEN adolescent cross sectional observational study

**DOI:** 10.1186/s12966-024-01678-4

**Published:** 2024-11-29

**Authors:** Ranjit Mohan Anjana, Harish Ranjani, Ester Cerin, Muhammad Akram, Jo Salmon, Terry L. Conway, Kelli L. Cain, Rajendra Pradeepa, Anthony Barnett, Cindy H. P. Sit, Delfien Van Dyck, Adriano Akira Hino, Andreia Pizarro, Adewale L. Oyeyemi, Wan Abdul Manan Wan Muda, Mika R. Moran, Jens Troelsen, Josef Mitáš, M. Zakiul Islam, Ana Queralt, Viswanathan Mohan, Erica Hinckson, James F. Sallis

**Affiliations:** 1 Dr. Mohan’s Diabetes Specialities Centre and Madras Diabetes Research Foundation, Chennai, Tamil Nadu India; 2 Mary MacKillop Institute for Health Research, Australian Catholic University, Melbourne, Australia; 3 School of Public Health, The University of Hong Kong, Hong Kong, China; 4 Institute for Physical Activity, Deakin University, Melbourne, Australia; 5 Herbert Wertheim School of Public Health, University of California San Diego, La Jolla, USA; 6 Department of Sports Science and Physical Education, The Chinese University of Hong Kong, Hong Kong, China; 7 Department of Movement and Sport Sciences, Faculty of Medicine and Health Sciences, Ghent University, Ghent, Belgium; 8 Health Sciences Postgraduate Program, Pontifícia Universidade Católica Do Paraná, Curitiba, Brazil; 9 Research Centre in Physical Activity, Health and Leisure, Faculty of Sport, University of Porto, Porto, Portugal; 10 Laboratory for Integrative and Translational Research in Population Health, Porto, Portugal; 11 College of Health Solutions, Arizona State University, Phoenix, AZ USA; 12 Graduate School of Public Health, Alma Ata University, Yogyakarta, Indonesia; 13 School of Public Health, Faculty of Social Welfare and Health Sciences, University of Haifa, Haifa, Israel; 14 Department of Sports Science and Clinical Biomechanics, University of Southern Denmark, Odense, Denmark; 15 Faculty of Physical Culture, Palacký University Olomouc, Olomouc, Czech Republic; 16 Department of Architecture, Bangladesh University of Engineering and Technology, Dhaka, Bangladesh; 17 Department of Nursing, University of Valencia, Valencia, Spain; 18 Department of Physical Activity and Nutrition, School of Sport & Recreation, Faculty of Health and Environmental Sciences, Auckland University of Technology, Auckland, New Zealand

**Keywords:** Built environment, Sedentary time, Youth, Social media, Screen time, Accelerometer

## Abstract

**Background:**

Understanding environmental correlates of sedentary behaviour (SB) among young people is important as such data can identify approaches to limit sedentary time. This paper estimates associations of parent-reported neighbourhood and adolescent-reported home environments with SB among adolescents aged 11–19 years from 14 countries.

**Methods:**

In the International Physical activity and the Environment Network (IPEN) Adolescent Study (an observational, cross-sectional multi-country study), adolescents wore a triaxial accelerometer for seven days that assessed sedentary time (ST). Adolescents completed survey measures of sedentary behaviour (SB) related to recreational screen time and sitting time in motor vehicles. Parents and adolescents completed surveys assessing neighbourhood and home environments. Accelerometer based ST was available in 3,982 adolescents while survey data were available for 6,302 dyads. We estimated the total and direct effects of each environmental attribute on ST and SB. Sex of the adolescent and city/country were examined as moderators.

**Results:**

The average ST in adolescents from 14 countries ranged from 7.8 to 10.5 h/day. Personal social media was the only significant correlate of total ST across both sexes. With respect to self-reported SB, adolescents accumulated an average of 3.8 h of non-school screen time per day and nearly 40 min of transport-related sitting time. Screen time was associated with all home environment variables, including social media account, as well as land use mix—diversity, traffic safety, and crime safety. Transport-related sitting time was related to land use mix—diversity, recreation facilities, walking facilities, and pedestrian infrastructure, but no home environment variables. City/country and sex were significant moderators of several associations.

**Conclusions:**

Both home and neighbourhood environment features were related to ST and SB. Having social media accounts emerged as a major contributor towards sedentarism in adolescents.

**Supplementary Information:**

The online version contains supplementary material available at 10.1186/s12966-024-01678-4.

## Background

Widespread access to a multitude of online content may be creating new media consumption patterns in adolescents that are associated with poor cardiometabolic [1] and mental [2] health. There is increased urgency to identify modifiable correlates of youth sedentary behaviour (SB) in its many forms, because youth SB increased and physical activity decreased during the COVID-19 pandemic [3–5]. SB refers to activities with low energy expenditure, typically characterized by sitting or reclining postures [6]. SB includes recreational activities such as watching television, using electronic devices such as computers or smartphones, playing video games, and riding in motorized vehicles [6, 7]. SB in adolescents has been linked to higher age, higher socioeconomic class, higher maternal education, living in a rural area, experimenting with alcohol, insufficient physical activity, and overweight [8]. Screen time was found to be similar for girls and boys [9].

SB in youth has been associated with neighbourhood and home environment features, such as the built environment (e.g., walkability, access to recreation facilities), transportation infrastructure, school environment, social and cultural factors, parental rules, and access to electronic devices [10–12]. These social and built environment variables interact with individual factors, such as adolescents’ personal preferences, motivation, and parents’ perceptions, in explaining adolescent SB [8, 9]. The home environment and social influences of parents have important roles in shaping SB and physical activity of youth, who may have limited behavioural and mobility autonomy, and thus may be particularly influenced by their daily environments [13].

Few studies have examined both home and neighbourhood environment correlates of adolescent SB. Most studies of environmental correlates of SB were conducted in relatively homogenous environments and populations and in one or a few cities from high-income countries. Because correlates of youth SB likely vary by type of SB and measurement methods [14], it is important to report findings for both device-based (objective) sedentary time (ST) and reported measures of SB. The present paper aims to address these gaps and advance evidence of associations of reported home and neighbourhood environment attributes with multiple indicators of SB among adolescents aged 11–19 years from 14 diverse countries. Potential moderating roles of sex and city/country were also examined.

## Methodology

### Study design

The International Physical activity and the Environment Network (IPEN) Adolescent study was an observational, cross-sectional, multicountry study with purposive sampling. IPEN study design aimed to include a broad range of built environment attributes both within and across country sites, and avoid confounding of built environments with neighbourhood income/SES. Systematic methods were used across countries to recruit participants who lived in areas reflecting broad variability in GIS-based walkability features and administrative units/census-based income/socioeconomic status. Within each country site, approximately equal numbers of participants were recruited from neighbourhoods in one of four systematically defined quadrants: low-walkability/low-income, low-walkability/high-income, high-walkability/low-income, high-walkability/high-income. The purposive sampling also involved recruiting approximately equal numbers of girls and boys and comparable age ranges across quadrants [15]. A coordinating centre based in San Diego, United States of America (USA) was responsible for monitoring comparability of methods, ensuring quality of all variables, and pooling data across countries. The overall design, methods, variations in measurements, and constraints of collecting international data were similar to those in an earlier IPEN study with adults [16].

### Setting

Briefly, adolescents, aged 11–19 years, along with one parent/guardian from 15 geographically and culturally diverse countries across six continents were recruited. Of the 14 countries examined in present analyses, nine were high-income (HIC), namely Australia (AUS, Melbourne), USA (Baltimore and Seattle regions), Belgium (BEL, Ghent), Czech Republic (CZE, Olomouc, Hradec Králové); Denmark (DNK, Odense); China (CHN, Hong Kong), Spain (ESP, Valencia), Israel (ISR, Haifa), and Portugal (PRT, Gondomar, Matosinhos, Maia, Porto, Valongo). Three countries were middle income: Malaysia (MYS, Kuala Lumpur), Brazil (BRA, Curitiba), and India (IND, Chennai). Two countries were low-income: Bangladesh (BGD, Dhaka) and Nigeria (NGA, Gombe). Data were also collected in New Zealand, but parent-reported surveys used in present analyses were not available. Participants were recruited from neighbourhoods or via schools to ensure they lived in administrative units (AUs; e.g., census blockgroups, meshblocks; termed ‘areas’) that varied in walkability and socioeconomic status (SES), with the aim of ensuring variability of environments and participants both within- and between-cities.

Detailed information regarding study sites, protocol, design, measures, and recruitment was reported previously [15]. The SES of areas was classified as low or high based mainly on country-specific publicly-available socio-demographic data. India and Nigeria categorised their AUs as low or high income based on investigator judgments, due to lack of reliable data sources. The city/region-specific area-level walkability and SES measures used were described previously [15].

All participants provided consent (parents/caretakers) and assent (adolescents). Study protocols in each country were approved by their Institution’s Ethics Committees. Participants’ confidentiality for pooled data was maintained by de-identifying individual records prior to transferring data to the Coordinating Center and using study identity (ID) numbers.

### Dependent variables

The dependent measures for present analyses (See Table 1 for details and supporting references) were adolescents’ self-reported screen time (min/day) and transport-related sitting time (min/day), and accelerometer-assessed sedentary time (min/day), during out-of-school periods on school days, on non-school days, and daily total.

**Table 1:** Description of the study measures (with references and scale/item details)

Measure types	References for methods, reliability, validity	Description	Scales/Items (response options; scoring)
**Dependent (Outcomes)**			
Sedentary time (ST) (accelerometer-measured)	Cain KL et al.;2021 [15] Cain KL et al.;2013 [17] Cain KL et al.;2018 [18] Evenson KR et al.;2008 [19]	Models used: Actigraph GT model GT1M, GT3X, or GT3X + (13 countries), 7164 (1 country) Epoch: 30 s Nonwear definition: > 60 min consecutive ‘0’ counts Valid day: 8 + hrs of wearing from 6AM—12AM Sedentary cut point: ≤ 100 counts per minute during valid wearing time Requested wear time: 7 days, with 4 + valid days required for inclusion Data processing software: MeterPlus v5.0	Accelerometer ST measures during 3 periods: - All valid wearing time across all valid days - Out-of-school on School days only - Non-school days only (scores indicate average minutes per day)
Sedentary behaviours (SB) (adolescent reported)	Rosenberg D et al.;2010 [20] Norman GJ et al.;2005 [21] Sallis JF et al.;2020 [22] Cerin E et al.;2017 [23] Cain KL et al.;2013 [17] Cerin E et al.;2014 [24]	Adolescents reported time spent in four SB’s on a typical school day (non-school hours), including 3 items indicating screen time (watching TV/DVDs/videos, playing sedentary video/computer games, using the internet/emailing/other electronic media for leisure), and 1 item indicating time riding in a motor vehicle.	Two SB measures: - *Screen time* minutes per day (sum of responses to 3 items: watching TV/DVDs/videos, playing sedentary video/computer games, and using the internet/emailing/other electronic media for leisure each day) - *Time riding in a motor vehicle* (single item) (all items had 6-category response options ranging from 0 min to 4 + hours, with hours converted to minutes per day)
**Independent variables**			
Neighborhood environment attributes (parent reported)	Cerin E et al.;2019 [25] Rosenberg D et al.;2009 [26]	IPEN-Adolescent adapted and validated version of the Neighborhood Environment Walkability Scale for Youth (NEWS-Y-IPEN) was completed by parents to assess perceived neighbourhood attributes.	Ten NEWS-Y-IPEN scales: - *Residential density scale* (weighted sum of 6 items, with scores ranging from 0–1048 reflecting low-to-high density) Four scales were reported walking time from home to various land uses: - *Land use mix—diversity* (13 items) - *Recreational facilities* (9 items) - *Parks-proximity* (2 items) - *Transit-stop proximity* (1 item) (scores above were averaged item responses using 5-point categories from > 30-min walk coded 1 [low proximity] to ≤ 5-min walk coded 5 [high proximity]) Five scales assessed neighborhood attributes for: *- Accessibility and walking facilities* (5 items; e.g., sidewalks, street crossings) *- Aesthetics* (3 items; e.g., nature, attractive buildings) *- Traffic safety* (3 items; e.g., speed of traffic, difficult to walk due to traffic) *- Pedestrian infrastructure and safety* (3 items; e.g., crosswalks and signals, good lighting) *- Safety from crime* (4 items; e.g., crime rate in area, stranger danger) (scores above were averaged item responses using 4-category options from strongly disagree coded 1 to strongly agree coded 4)
Home electronics/media environment (adolescent reported)	Rosenberg D et al.;2010 [20] Norman GJ et al.;2005 [21] Sallis JF et al.;2020 [22] Cerin E et al.;2017 [23] Cain KL et al.;2013 [17]	Ten survey items to assess adolescent's home electronics/media environment, drawn from prior studies that provided evidence of good test–retest reliability and associations with SB.	Three home environment measures: *Electronic devices in the bedroom (6 items assessed devices in the adolescent’s bedroom:* television, computer, video game system (non-hand-held—Playstation, Xbox), music player (radio, CD player, stereo), DVD player, and internet access) *Personal electronic devices (3 items assessed portable devices “for your own use”:* cell phone, hand-held video game player (Game Boy, Sony PSP), and personal audio device (iPod, MP3 player)). *Has own social media (a single item assessed whether adolescents had their own social media account* (e.g., Facebook, Instagram).

### Sedentary time (ST) assessed by accelerometery

To assess ST, adolescents wore ActiGraph accelerometers. Thirteen countries used a GT model (GT1M, GT3X, or GT3X +), and the USA primarily used the older 7164 model. The low frequency extension (LFE) filter, which improves comparability between data collected with various models [17], was activated in 12 countries that used a GT model, although one of these countries used the LFE for only about half of their sample. Participants were instructed to wear the accelerometer on a belt around the waist during waking hours (except when bathing or swimming) for at least 7 days. Accelerometer vertical-axis data were collected with (or converted to) a 30-s epoch. Accelerometer data from all countries were sent to the Coordinating Center where trained researchers screened and scored all data using standard protocols and MeterPlus v.5.0 software.

Three estimates of ST were computed and analysed: average min/day across all valid days (total ST), average of out-of-school periods on school days, and average of non-school days. We refer to “non-school” days instead of “weekends”, because school days varied across countries. See Table 1 for a summary of accelerometer methods. Detailed accelerometer protocols can be found on the IPEN website at https://ipenproject.org/resource-hub/resources/accelerometers/.

### Sedentary behaviours (SB’s) assessed by self-report

Adolescents reported time spent in four SB’s on a typical school day (non-school hours): watching Television (TV)/ Digital Versatile Disc (DVDs)/videos, playing sedentary video/computer games, using the internet/emailing/other electronic media for leisure, and riding in a motor vehicle. Recreational screen time (minutes (min)/day) was constructed by summing the first three items above. Time riding in a motor vehicle (min/day) was retained as a separate variable.

### Independent variables: built and social environment attributes

Independent variables were parental reports of neighbourhood/community environments and adolescent reports of home environments (See Table 1 for details and supporting references). Surveys were translated to the local language (if needed), back-translated to English, verified by the Coordinating Center, then pilot tested in each country before making final adaptations.

#### Parent-reported

The Neighbourhood Environment Walkability Scale for Youth (NEWS-Y) [25, 26] was completed by parents to measure perceived neighbourhood attributes hypothesized to influence physical activity and ST in adolescents. NEWS-Y was culturally adapted as needed for international use. To help standardise scoring of pooled international data, a scoring protocol for NEWS-Y items common to all IPEN Adolescent countries was developed and validated (NEWS-Y-IPEN) [25]. Ten summary scores were computed, as shown in Table 1. Parent-reported NEWS-Y summary scales have good evidence of test–retest reliability, associations with adolescent-reported scores, construct validity based on associations with adolescent physical activity [26], and criterion validity based on Geographic Information Systems measures of numerous NEWS scales [27]. Use of parent reports of NEWS-Y neighbourhood environment scales was also justified by limiting the respondent burden for adolescents who did not complete the entire NEWS-Y.

#### Adolescent-reported

To assess the home electronics environment, adolescents completed 10 items, with evidence of test–retest reliability and associations with SB [20–23]. The three home environment measures were electronic devices in the bedroom (six items), personal electronic devices (three items), and own social media account (one item), as shown in Table 1.

### Data analysis

Separate analyses were conducted on the whole sample with self-report data on SB (*N* = 6,302) and the subsample with accelerometry-assessed ST (*n* = 3,982). Descriptive statistics (e.g., means, standard deviations, frequencies, percentage of missing values) were computed for the two pooled samples and by city (within each sample). As 21.1% cases had missing data on at least one of the variables included in the regression models, and data were not completely missing at random, main regression analyses were conducted on 20 imputed datasets created using multiple imputations by chained equations and accounting for clustering at the school and administrative unit levels. Multiple imputations were performed using the package ‘mice’ in R [28] according to van Burren’s model-building recommendations [29]. For comparison purposes, analyses were also conducted on cases with complete data (*N* = 4,975 with survey only and *n* = 3,148 with accelerometers) and reported in the supplementary material.

We adopted a causal inference approach to our analyses and used directed acyclic graphs (DAGs) (reported in Supplementary Materials, Figures S1, S2, S3) to inform the selection of a minimal sufficient set of confounders and other covariates (mediators) to be included in the regression models estimating the total and direct effects of each environmental attribute on SB outcomes. Here, the meaning of ‘effect’ needs to be interpreted in the context of the cross-sectional observational nature of the study with possible unmeasured confounders. In the context of this study, ‘total’ effects refer to confounder-adjusted associations between exposures (perceived environmental attributes) and SB outcomes unadjusted for potential environmental mediators. ‘Direct’ effects refer to associations between exposures and outcomes adjusted for potential confounders as well as environmental mediators. We hypothesised that densification (represented by perceived residential density) would potentially shape most of the other neighbourhood characteristics, including land use mix – diversity, recreation facilities, accessibility and walking facilities, traffic safety, pedestrian infrastructure and safety, park proximity and transit stop proximity [30], which would then act as potential mediators of the associations between residential density and SB outcomes. We also hypothesised that the availability of electronic devices in the bedroom, personal devices and own social media accounts (classified as home environment variables) would be in part determined by the extent to which parents perceived the neighbourhood to be activity friendly. Hence, we treated them as mediators of the associations of all neighbourhood environmental attributes with screen time and ST. However, as these three home environment variables are unlikely to influence transport-related sitting, they were excluded from models of transport-related sitting. From the above, it follows that the covariates differed across models, as specified in Supplementary Materials (see Tables S1, S5, S9).

The meaning of ‘effect’ needs to be interpreted in the context of the cross-sectional observational nature of the study with possible unmeasured confounders. A ‘total effect’ refers to the total extent to which an outcome is potentially affected by an exposure, while a ‘direct effect’ represents the effect of an exposure on the outcome adjusted for potential mediators included in the regression models. Multicollinearity was assessed by computing the Variance Inflation Factor (VIF) for each variable included in the models.

Generalised additive mixed models [GAMMs; package ‘mgcv’ version 1.8.34 [31]] with random intercepts at the administrative unit and school levels were used to estimate environment-outcome total and direct associations. GAMMs are generalised linear mixed models (GLMMs) in which the outcome variable may depend on unknown smooth (curvilinear) functions of one or more explanatory variables. GAMMs are more flexible than GLMMs because they allow modelling and testing for curvilinear relationships (if any) of unknown form. Screen time and three accelerometer-based measures of ST were approximately normally distributed and were modelled using GAMMs with Gaussian variance and identity link functions. Transport-related ST was positively skewed and modelled using gamma variance and logarithmic link functions. Smooth terms (thin plate splines) were used to model curvilinear associations, and evidence of curvilinearity was based on the comparison of Akaike Information Criterion (AIC) values from models with smooth vs. linear terms (10-unit difference in AIC) [32]. Moderating effects of adolescent sex and study site (cities) on environment-outcome associations were estimated by adding two-way interaction terms to the corresponding main effect GAMMs. Statistically significant interaction effects were probed by estimating sex-specific and/or site-specific associations.

## Results

### Descriptive statistics

Table 2 describes the complete study sample (*n* = 6,302), and Table S1 (Supplementary Material 1) reports results for the sub-sample with accelerometer-assessed ST (*n* = 3,982). Adolescents’ average age across sites ranged from 13.4 to 16.6 years. Substantial between-site differences were observed for highest education in the household, social media account, and electronic devices in the home. For example, adolescents from India had an average of 1.2 electronic devices in the bedroom and 0.5 personal electronic devices, while the average number of such devices in Denmark was 4.2 and 2.3, respectively. In India and Bangladesh, fewer than 30% of adolescents reported having their own social media account, compared to higher SES countries where it was over 90% (Tables 2* and S1, Supplementary Material 1*).

**Table 2:** Overall and city-specific sample characteristics (*N* = 6302; whole sample)

		**High-income countries**											**Low-middle-income countries**				
	**All sites**	**Melb** **AUS**	**Ghent** **BEL**	**Hradec Kralove** **CZE**	**Olomouc** **CZE**	**Odense** **DNK**	**Hong Kong** **CHN**	**Haifa** **ISR**	**Various cities** **PRT**	**Valencia** **ESP**	**Baltimore** **USA**	**Seattle** **USA**	**Dhaka** **BGD**	**Curitiba** **BRA**	**Chennai IND**	**Kuala Lumpur** **MYS**	**Gombe** **NGA**
**N**	6302	438	291	155	183	210	1295	232	184	465	485	443	92	493	316	752	268
***Socio-demographics***																	
**Child’s age (years)**																	
Mean (SD)	14.5 (1.7)	14.9 (1.6)	13.4 (1.4)	14.3 (1.7)	13.7 (1.6)	13.0 (1.2)	14.3 (1.7)	15.3 (1.4)	15.9 (1.2)	16.6 (0.8)	14.2 (1.4)	14.0 (1.4)	13.9 (1.8)	14.1 (1.6)	13.8 (1.5)	14.4 (1.3)	15.3 (1.6)
**Child’s sex**																	
% male	46.5	40.2	41.9	50.3	44.8	41.0	42.8	39.2	37.5	44.9	46.6	52.8	53.3	48.9	52.5	53.1	54.5
**Highest education in household**																	
% College or higher % missing	46.7 11.2	35.4 45.7	73.5 1.7	32.3 36.1	15.9 54.1	66.7 3.3	35.3 0.0	60.8 1.3	35.9 18.5	55.7 0.0	73.8 1.0	76.1 0.2	58.7 0.0	40.8 0.0	47.2 0.3	24.9 38.2	53.7 3.4
**Area-level SES**																	
% High	47.1	45.9	51.9	49.7	44.3	53.8	46.5	50.4	52.7	53.5	50.9	49.2	44.6	43.2	48.1	39.9	41.0
***Adolescent-reported home environment***																	
**Electronic devices in the bedroom [0–6]**																	
Mean (SD) % missing	2.5 (1.6) 4.3	2.7 (1.5) 0.5	2.5 (1.5) 0.5	3.6 (1.4) 0.0	3.4 (1.4) 0.0	4.2 (1.4) 0.1	1.9 (1.5) 1.2	3.0 (1.5) 0.0	3.4 (1.6) 0.1	2.8 (1.5) 0.0	2.8 (1.7) 0.02	2.4 (1.6) 0.02	2.0 (1.6) 0.0	2.6 (1.6) 0.0	1.2 (1.4) 1.6	2.0 (1.6) 0.3	2.0 (1.7) 0.0
**Personal electronic devices [0–3]**																	
Mean (SD) % missing	1.8 (0.9) 2.7	2.0 (0.7) 0.9	2.0 (0.7) 0.5	1.9 (0.7) 0.0	1.7 (0.7) 0.0	2.3 (0.7) 0.1	1.8 (0.8) 1.2	1.6 (0.7) 0.0	2.2 (0.8) 0.1	2.3 (0.7) 0.0	2.2 (0.8) 0.02	2.2 (0.8) 0.02	1.0 (0.9) 0.0	1.3 (0.7) 0.0	0.5 (0.8) 0.0	1.6 (0. 9) 0.3	1.1 (0.8) 0.0
**Having own social media**																	
% Yes % missing	76.1 2.7	84.7 6.9	67.7 10.3	91.0 0.0	89.6 0.0	76.2 3.8	86.3 5.8	86.6 0.0	90.2 2.2	82.6 0.0	71.1 0.2	67.7 0.2	29.4 1.1	92.9 0.0	27.8 0.0	75.7 2.8	59.3 0.0
***Parent-reported neighbourhood environment***																	
**Residential density [0–1000]**																	
Mean (SD) % missing	213.5 (220) 14.8	48.9 (103.8) 3.0	71.6 (109.0) 0.03	155.8 (114.6) 0.8	111.8 (98.4) 1.4	106.1 (110.0) 0.0	468.4 (203.2) 0.0	216.8 (148.5) 0.02	121.6 (94.0) 0.8	251.1 (134.7) 0.0	38.7 (57.9) 1.2	23.8 (33.7) 0.6	177.3 (82.9) 0.0	96.3 (126.7) 0.0	65.6 (77.8) 0.0	292.0 (230.4) 6.7	271.5 (159.4) 0.2
**Land use mix-diversity**^**a**^ [1-5]																	
Mean (SD) % missing	3.2 (0.8) 12.6	3.0 (0.8) 3.0	3.4 (0.83) 0.1	3.3 (0.9) 0.8	3.1 (0.9) 1.4	3.0 (0.9) 0.0	3.4 (0.8) 0.0	3.0 (0.9) 0.0	3.5 (0.8) 0.8	4.2 (0.5) 0.0	2.7 (0.9) 0.03	2.7 (0.9) 0.0	3.4 (0.7) 0.02	3.0 (0.7) 0.0	3.4 (0.7) 0.0	2.7 (0.8) 6.3	3.4 (0.8) 0.1
**Recreation facilities**^**b**^ [1-5]																	
Mean (SD) % missing	2.7 (0.9) 12.6	2.8 (0.9) 2.97	2.7 (0.9) 0.1	3.0 (0.9) 0.84	2.9 (0.9) 1.4	3.7 (0.7) 0.0	2.8 (0.9) 0.0	2.4 (0.8) 0.0	2.6 (0.9) 0.8	2.9 (0.8) 0.0	2.9 (0.9) 0.03	2.9 (0.8) 0.0	2.0 (0.7) 0.02	2.4 (0.8) 0.0	1.8 (0.6) 0.0	2.3 (0.9) 6.25	2.8 (0.5) 0.1
**Accessibility and walking facilities ** [1-4]																	
Mean (SD) % missing	3.0 (0.5) 12.6	3.3 (0.5) 3.1	3.0 (0.6) 0.1	3.2 (0. 5) 0.9	3.1 (0.5) 1.5	3.1 (0.5) 0.0	3.0 (0.5) 0.0	3.1 (0.5) 0.02	3.0 (0.4) 0. 8	3.6 (0.4) 0.0	3.0 (0.6) 0.02	2.8 (0.7) 0.0	2.8 (0.6) 0.0	2.9 (0.6) 0.0	2.6 (0.6) 0.0	2.8 (0.4) 6.2	2.7 (0.7) 0.1
**Aesthetics ** [1-4]																	
Mean (SD) % missing	2.5 (0.8) 12.7	3.0 (0.8) 3.1	2.3 (0.7) 0.1	2.3 (0.6) 0.9	2.2 (0.7) 1.5	2.7 (0.8) 0.0	2.5 (0.7) 0.0	2.6 (0.8) 0.02	2.4 (0.5) 0.8	2.3 (0.7) 0.0	3.0 (0.7) 0.02	3.1 (0.7) 0.0	1.8 (0.8) 0.0	2.4 (0.9) 0.0	1.5 (0.8) 0.0	2.5 (0.6) 6.2	2.9 (0.9) 0.1
**Traffic safety ** [1-4]																	
Mean (SD) % missing	2.6 (0.6) 12.6	2.9 (0.6) 3.1	2.5 (0.6) 0.1	2.9 (0.6) 0.8	2.8 (0.6) 1.4	2.9 (0.7) 0.0	2.8 (0.5) 0.0	2.4 (0.7) 0.0	2.8 (0.5) 0.7	2.6 (0.7) 0.0	2.5 (0.6) 0.02	2.7 (0.6) 0.0	2.4 (0.6) 0.0	2.2 (0.8) 0.0	2.3 (0.9) 0.0	2.4 (0.5) 6.2	3.0 (0.9) 0.2
**Pedestrian infrastructure & safety ** [1-4]																	
Mean (SD) % missing	2.9 (0.6) 12.6	2.8 (0.6) 3.1	2.7 (0.6) 0.1	3.0 (0.6) 0.8	2.9 (0.5) 1.4	2.9 (0.7) 0.0	3.0 (0.6) 0.0	2.9 (0.7) 0.0	2.9 (0.5) 0.7	3.0 (0.62) 0.0	2.8 (0.7) 0.03	2.9 (0.6) 0.0	2.5 (0.6) 0.0	2.6 (0.8) 0.0	2.9 (0.8) 0.0	2.7 (0.6) 6.2	3.0 (0.8) 0.2
**Crime safety ** [1-4]																	
Mean (SD) % missing	2.8 (0.9) 12.6	3.1 (0.8) 3.1	3.1 (0.8) 0.1	2.9 (0.7) 0.9	2.8 (0.7) 1.4	3.7 (0.6) 0.0	2.7 (0.9) 0.0	3.3 (0.9) 0.03	3.0 (0.6) 0.8	3.3 (0.8) 0.02	3.0 (0.7) 0.03	3.1 (0.7) 0.0	2.0 (0.9) 0.0	2.0 (0.4) 0.0	3.0 (1.1) 0.0	2.0 (0.7) 6.2	2.7 (1.2) 0.1
**Transit stop proximity ** [1-5]																	
Mean (SD) % missing	4.1 (1.1) 12.9	4.5 (0.8) 3.0	4.6 (0.8) 0.2	4.3 (1.1) 0.8	4.3 (1.0) 1.4	4.6 (0.7) 0.0	3.9 (1.1) 0.0	4.5 (0.9) 0.03	4.6 (0.8) 0.8	4.8 (0.6) 0.0	3.6 (1.4) 0.03	4.0 (1.2) 0.0	2.1 (1.3) 0.1	4.7 (0.6) 0.0	3.8 (1.1) 0.02	3.2 (1.4) 6.3	4.0 (1.0) 0.2
**Park proximity ** [1-5]																	
Mean (SD) % missing	3.0 (1.2) 13.2	3.8 (1.0) 3.0	3.1 (1.3) 0.1	3.0 (1.1) 0.8	2.3 (1.2) 1.5	3.3 (1.3) 0.0	3.4 (1.1) 0.0	3.2 (1.2) 0.0	2.9 (1.0) 0.8	4.0 (0.8) 0.0	2.9 (1.2) 0.03	3.1 (1.2) 0.0	1.8 (0.9) 0.1	2.7 (1.0) 0.0	2.2 (1.1) 0.0	2.5 (1.1) 6.8	1.0 (0.0) 0.0
***Sedentary behaviour***																	
**Screen time (min/day)**																	
Mean (SD) % missing	228 (159) 1. 6	253 (155) 0.5	207 (144) 0.5	229 (148) 0.0	241 (156) 0.0	224 (142) 0.2	189 (141) 0.0	241 (145) 0.02	181 (143) 0.1	203 (124) 0.0	234 (166) 0.02	186 (135) 0.02	169 (132) 0.0	333 (167) 0.0	145 (102) 0.0	326 (171) 0.3	170 (161) 0.0
**Transport-related sitting time (min/day)**																	
Mean (SD) % missing	37.9 (46.0) 2.1	55.2 (58.2) 0.5	32.2 (44.7) 0.6	26.2 (30.8) 0.0	27.1 (37.5) 0.0	15.8 (29.4) 0.2	41.6 (49.9) 0.0	53.6 (49.3) 0.02	35.1 (33.8) 0.1	14.5 (23.3) 0.0	51.7 (47.9) 0.02	45.3 (40.0) 0.02	16.5 (30.8) 0.0	30.9 (35.3) 0.0	24.4 (45.9) 0.0	48.6 (54.5) 0.7	29.3 (48.7) 0.1

*Melb* Melbourne, *SES* Socio-economic-status, *SD* Standard deviation, *min* minutes, *AUS* Australia, *BEL* Belgium, *CZE* Czech Republic, *DNK* Denmark, *HKG* Hong Kong, *CHN* China, *ISR* Israel, *PRT* Portugal, *ESP* Spain, *USA* Unites States of America, *BGD* Bangladesh, *BRA* Brazil, *IND* India, *MYS* Malaysia, *NGA* Nigeria

^a^Excluding transit stops

^b^Excluding parks

Between-site variability was also evident in parent-reported aspects of the neighbourhood environment, particularly in relation to residential density, with less-populous countries having lower scores compared to more-populous countries like China. Bangladesh and India had low average scores for aesthetics, while Denmark, USA, and Australia scored higher on this variable. Australia had one of the highest reported access to parks, while Nigerian parents reported no access (i.e., > 30-min walk), and parents in Bangladesh and India reported poor access to parks. All sites, except Bangladesh, reported relatively good access (10–20-min walk) to transit stops (transit stop proximity). Similarly, on average, all sites reported good pedestrian infrastructure, accessibility and walking facilities. Average scores on the traffic safety subscale indicated potential parental concerns about traffic in Brazil, Malaysia, Bangladesh, India, and Israel, and concerns about crime in the first three countries.

With respect to self-reported SB, adolescents accumulated an average of 3.8 h of non-school screen time per day and nearly 40 min of transport-related sitting time (Table 2). Bangladesh and India had among the lowest levels of screen time (< 3 h/day), and Brazil and Malaysia among the highest (> 5 h (hr)/day). Transport-related sitting was among the highest in USA, exceeding 50 min/day, and the lowest was in Spain, with less than 20 min/day. On an average day, adolescents spent 8.9 of 13.5 h (i.e., 66.1%) of accelerometer wear-time being sedentary (Table S1; Supplementary Material 1). They spent 65.5% and 63.8% of wear-time being sedentary on non-school days and during non-school periods on school days, respectively. The highest wear-time being sedentary on out-of-school periods on school days and non-school days was seen in Spain (69.4%, 72.5% respectively) and the lowest in Nigeria (57.8%, 69.4% respectively).

### Perceived environment correlates of sedentary behaviour

#### Neighbourhood environment and screen time

Table 3 reports the pooled total and direct effects of perceived home and neighbourhood environmental attributes on adolescents’ self-reported screen time. Parent-reported neighbourhood land use mix–diversity, traffic safety, and crime safety were negatively related to adolescent screen time. Total effects of these neighbourhood characteristics were slightly stronger than direct effects, indicating results were in part mediated by other environmental variables (i.e., electronic devices in the home; own social media). Although the pooled associations of neighbourhood residential density and park proximity with screen time were not statistically significant (Table 3), adolescent sex moderated these associations (Residential density by Sex interaction: b_Total&Direct_ = 0.07; 95% CI: 0.04, 0.11; p < 0.001; Park proximity by Sex interaction: b_Direct_ = 6.16; 95% CI: 0.05, 12.26; *p* = 0.048). Males showed significant negative associations of these two neighbourhood characteristics with screen time (Residential density: b_Total&Direct_ = -0.05; 95% CI: -0.09, -0.02; *p* = 0.003; Park proximity: b_Direct_ = -6.05; 95% CI: -11.87, -0.22; *p* = 0.044) but females did not (Residential density: b_Total_ = 0.02; 95% CI: -0.01, 0.05; *p* = 0.228; Parks: b_Direct_ = 0.11; 95% CI: -5.25, 5.48; *p* = 0.967).

**Table 3:** Total and direct effects of perceived neighbourhood and home environment characteristics on adolescents’ screen time and transport-related sitting time (*N* = 6,302)

Characteristic [range of values]	Effect	Screen time (min/day)			Transport-related sitting time (min/day)		
		*b*	95% CI	*p*	e^*b*^	95% CI	*p*
*Parent-reported neighbourhood environment*							
Residential density [0–1000]	Total	-0.01	-0.04, 0.01	.339	0.9998	0.9996, 1.0000	.073
	Direct	-0.02	-0.05, 0.01	.241	0.9999	0.9997, 1.0001	.288
Land use mix – diversity [1–5]	Total	-6.35	-11.41, -1.29	.014	0.90	0.86, 0.93	< .001
	Direct	-6.76	-12.68, -0.84	.026	0.90	0.86, 0.94	< .001
Transit stop proximity [1–5]	Total	3.04	-2.29, 8.38	.268	1.00	0.96, 1.06	.738
	Direct	5.75	-0.20, 11.70	.065	1.00	0.96, 1.00	.738
Recreation facilities [1–5]	Total	-3.20	-8.18, 1.79	.210	0.96	0.92, 1.00	.050
	Direct	-0.92	-6.54, 4.71	.750	0.96	0.92, 1.00	.050
Park proximity [1–5]	Total	-3.64	-7.54, 0.26	.069	1.00	0.97, 1.03	.888
	Direct	-2.79	-7.46, 1.88	.244	1.00	0.97, 1.03	.888
Accessibility & walking facilities [1–4]	Total	-1.70	-9.14, 5.74	.655	0.91	0.86, 0.97	.003
	Direct	1.41	-6.35, 9.17	.722	0.91	0.86, 0.97	.003
Traffic safety [1–4]	Total	-15.32	-21.98, -8.66	< .001	0.97	0.92, 1.02	.299
	Direct	-12.35	-19.18, -5.52	< .001	0.97	0.92, 1.02	.299
Pedestrian infrastructure & safety [1–4]	Total	-0.92	-7.47, 5.62	.783	0.95	0.90, 0.998	.040
	Direct	3.89	-3.23, 11.00	.286	0.95	0.90, 0.998	.040
Crime Safety [1–4]	Total	-7.90	-12.85, -2.96	.002	0.98	0.94, 1.02	.243
	Direct	-6.28	-11.13, -1.42	.012	0.98	0.94, 1.02	.243
Aesthetics [1–4]	Total	-5.23	-10.84, 0.37	.068	1.01	0.96, 1.06	.738
	Direct	-3.37	-9.14, 2.40	.253	1.01	0.96, 1.06	.738
*Adolescent-reported home environment*							
Electronic devices in the bedroom [0–6]	Total^a^	15.95	13.59, 18.30	< .001	-	-	-
Personal electronic devices [0–3]	Total^a^	26.58	21.97, 31.20	< .001	-	-	-
Having own social media [0–1]	Total^a^	37.20	27.28, 47.11	< .001	-	-	-

*b* regression coefficient, e^*b*^ exponentiated regression coefficient, *CI* confidence intervals, *p p*-value,—not estimates as theoretically unrelated to sedentary behaviour outcome

^a^Total and direct effects are equivalent as no mediating variables of characteristic-outcome associations were included in the models. All analyses were performed on 20 imputed datasets. Complete case analyses are in the Supplementary Material 2 (Tables S2 and S6). Model covariates are reported in Tables S1 and S5 (Supplementary Material 2). Statistically significant effects (*p* < .05) are in bold

#### Home environment and screen time

All home environment attributes examined in this study, including having own social media account, personal electronic devices, and electronic devices in the bedroom, were positively related to screen time in pooled analyses. While no between-sex differences were observed in these associations, study site was a significant moderator of the effects of electronic devices in the bedroom and personal social media on screen time. The associations across sites ranged from null to strongly positive (*Table S2, Supplementary Material 1*). Significant positive associations between personal social media and screen time were observed only in two HIC [Baltimore (USA) and Melbourne (Australia)] and four LMIC cities [Gombe (Nigeria), Curitiba (Brazil), Dhaka (Bangladesh) and Chennai (India)]. A weaker positive association was found in Olomouc (Czech Republic). As to electronic devices in the bedroom, location-specific associations were more consistent. Significant positive associations were found in 11 of the 16 study sites. Insufficient support of an association between electronic devices in the bedroom and screen time was observed in Spain, Portugal, Czech Republic, India and Israel.

#### Transport-related sitting time

Parent-reported land use mix–diversity, recreation facilities, accessibility and walking facilities, and pedestrian infrastructure and safety were negatively related to adolescents’ transport-related sitting time (Table 3). Parent-reported neighbourhood residential density was negatively associated with transport-related sitting time in females (e^b^ = 0.9997; 95% CI: 0.9994, 0.9999; *p* = 0.008) but not in males (e^b^ = 0.9999; 95% CI: 0.9997, 1.0002; *p* = 0.728). Study location moderated the total effects of several neighbourhood environment attributes (*Table S3,* Supplementary Material 1) and the direct effect of residential density on adolescents’ transport-related sitting time. For the latter, a negative association was observed only in Bangladesh (e^b^ = 0.996; 95% CI: 0.993, 0.999; *p* = 0.004), while no evidence of associations was found in other cities.

Statistically significant negative associations between land use mix–diversity and transport-related sitting time were observed in five cities (*Table S3,* Supplementary Material 1). However, four more cities had exponentiated regression coefficients smaller than 0.90, suggestive of a negative association. Denmark and China had statistically significant negative associations with adolescents’ transport-related sitting time for both access to walking facilities and pedestrian infrastructure safety, while Spain showed a negative association only for the latter attribute. In contrast, the associations of transport-related sitting time with pedestrian infrastructure and safety among adolescents from India and Nigeria were positive, as were those with neighbourhood aesthetics (Table S3, Supplementary Material 1). Adolescents in Hong Kong were the only ones to show a statistically significant negative association between parent-perceived traffic safety and transport-related sitting time. Although study location was a significant moderator of the association between park proximity and transport-related sitting time, no city-specific associations were statistically significant.

### Accelerometer-assessed sedentary time

Few significant associations were found between reported environment characteristics and adolescents’ accelerometer-assessed ST in the whole accelerometer sample (Table 4). Having a personal social media account was the only significant positive correlate of total ST. A personal social media account was positively related to ST during out-of-school periods on school days, and parent-reported access to recreation facilities was negatively related to ST during out-of-school periods on school days. No significant correlates of ST on non-school days were found, nor did the above associations differ significantly across study locations (Table 4).

**Table 4:** Total and direct effects of perceived neighbourhood and home environment characteristics on adolescents’ accelerometer-assessed sedentary time [multiple imputation analyses; *N* = 3982]

		Total sedentary time (min/day)			Sedentary time (min/day) during out-of-school periods on school days			Sedentary time (min/day) on non-school days		
Model	Effect estimated	*b*	95% CI	*p*-value	*b*	95% CI	*p*-value	*b*	95% CI	*p*-value
1 T	Total effects on Residential density	0.002	-0.01, 0.02	.744	0.005	-0.01, 0.01	.360	0.007	-0.01, 0.03	.493
1D	Direct effects of Residential density	0.003	-0.01, 0.02	.719	0.005	-0.01, 0.02	.313	0.005	-0.02, 0.02	.660
2 T	Total effects of Land use mix-diversity^a^	-1.16	-3.80, 1.49	.391	-1.06	-2.85, 0.72	.243	1.92	-1.55, 5.40	.279
2D	Direct effects of Land use mix diversity^a^	-0.56	-3.98, 2.86	.749	-0.41	-2.68, 1.86	.723	2.19	-2.30, 6.67	.341
3 T	Total effects of Transit stop proximity	-0.77	-2.72, 1.18	.438	-0.41	-1.74, 0.93	.549	-0.78	-3.49, 1.93	.573
3D	Direct effects of Transit stop proximity	-0.34	-254, 1.86	.764	0.08	-1.42, 1.57	.920	-1.64	-4.69, 1.40	.290
4 T	Total effects of Recreation facilities^b^	-1.54	-3.98, 0.91	.218	-1.69	-3.36, -0.01	.048	1.04	-2.44, 4.51	.558
4D	Direct effects of Recreation facilities^b^	-1.75	-4.91, 1.42	.280	-2.13	-4.27, 0.01	.052	0.43	-4.02, 4.88	.849
5 T	Total effects of Park proximity	-0.10	-2.00, 1.80	.920	0.14	-1.16, 1.45	.830	0.50	-2.09, 3.08	.705
5D	Direct effects of Park proximity	0.81	-1.47, 3.09	.488	1.25	-0.35, 2.84	.126	-0.02	-3.10, 3.06	.989
6 T	Total effects of Accessibility and walking facilities	-1.02	-4.61, 2.57	.578	-1.84	-4.33, 0.61	.140	1.88	-3.11, 6.86	.461
6D	Direct effects of Accessibility and walking facilities	-0.66	-4.62, 3.31	.746	-1.29	-3.97, 1.40	.348	1.85	-3.74, 7.44	.517
7 T	Total effects of Traffic safety	-1.00	-4.03, 2.04	.521	-1.33	-3.45, 0.80	.222	0.78	-3.44, 5.00	.718
7D	Direct effects of Traffic safety	-0.81	-3.98, 2.37	.619	-0.96	-3.17, 1.26	.397	0.86	-3.61, 5.34	.706
8 T	Total effects of Pedestrian infrastructure and safety	-0.55	-3.46, 2.35	.710	-1.74	-3.72, 0.25	.087	-0.05	-4.10, 4.00	.982
8D	Direct effects of Pedestrian infrastructure and safety	-0.11	-3.27, 3.06	.948	-1.21	-3.36, 0.93	.268	-0.71	-5.16, 3.74	.755
9 T	Total effects of Crime Safety	-0.80	-3.17, 1.57	.507	-0.40	-2.03, 1.24	.634	-0.31	-3.60, 2.98	.854
9D	Direct effects of Crime Safety	-0.70	-3.14, 1.74	.576	-0.08	-1.76, 1.61	.929	-0.43	-3.84, 2.98	.805
10 T	Total effects of Aesthetics	0.78	-1.89, 3.45	.567	0.18	-1.65, 2.01	.847	-0.16	-3.86, 3.54	.933
10D	Direct effects of Aesthetics	1.20	-1.60, 4.00	.401	0.80	-1.11, 2.71	.413	-0.51	-4.40, 3.37	.795
11 T	Total effects of Personal electronic devices	0.88	-1.52, 3.28	.473	0.89	-0.76, 2.53	.290	2.17	-1.20, 5.55	.207
11D	Direct effects of Personal electronic devices	0.88	-1.52, 3.28	.473	0.89	-0.76, 2.53	.290	2.17	-1.20, 5.55	.207
12 T	Total effects of Having own social media	5.07	0.12, 10.03	.045	3.87	0.47, 7.28	.026	5.21	-1.88, 12.30	.150
12D	Direct effects of Having own social media	5.07	0.12, 10.03	.045	3.87	0.47, 7.28	.026	5.21	-1.88, 12.30	.150
13 T	Total effects of Electronic devices in the bedroom	-0.07	-1.31, 1.17	.911	0.40	-0.45, 1.25	.351	0.77	-1.06, 2.61	.410
13D	Direct effects of Electronic devices in the bedroom	-0.07	-1.31, 1.17	.911	0.40	-0.45, 1.25	.351	0.77	-1.06, 2.61	.410

All analyses were performed on 20 imputed datasets. Complete case analyses in Tables S10, S12 and S14 (Supplementary Material 2). Model covariates are in Table S9 (Supplementary Material 2). Statistically significant effects (*p* < .05) are in bold

*b* regression coefficient, *CI* confidence intervals, *p* p-value

^a^Excluding transit stops

^b^Excluding parks

Adolescent sex moderated several associations between environment characteristics and ST (Table 5). Land use mix–diversity was negatively related to ST in females only, especially on non-school days. Parent-reported recreation facilities in the neighbourhood were also negatively related to accelerometry-assessed sedentary time in females only, particularly during out-of-school hours on school days. There were negative relations between transit stop proximity and females’ total ST and out-of-school ST on school days, which were attenuated after adjusting for home environment variables. While positive associations of accessibility and walking facilities with ST were found in males, particularly on non-school days, females showed negative associations, particularly during non-school hours on school days. Park proximity was unrelated to ST in females but positively related in males. Finally, while number of electronic devices in the bedroom was not significantly associated with ST in females, it was positively related to out-of-school ST on school days in males (Table 5).

**Table 5:** Adolescent sex-specific total and direct effects of perceived neighbourhood and home environment characteristics on adolescents’ accelerometry-assessed sedentary time (only significant moderating effects reported)

Characteristic [range of values]	Effect	Total sedentary time (min/day)			Sedentary time (min/day) during out-of-school periods on school days			Sedentary time (min/day) on non-school days		
		*b*	95% CI	*p*	*b*	95% CI	*p*	*b*	95% CI	*p*
**Associations in males**										
*Parent-reported neighbourhood environment*										
Land use mix – diversity [1–5]	Total	1.97	-1.64, 5.58	.284	-	-	-	1.00	-3.32, 5.32	.650
	Direct	2.63	-1.64, 6.89	.228	-	-	-	1.93	-2.45, 6.31	.388
Transit stop proximity [1–5]	Total	1.13	-1.50, 3.75	.401	1.29	-0.48, 3.06	.155	-	-	-
	Direct	1.65	-1.20, 4.50	.256	1.83	-0.08, 3.75	.061	-	-	-
Recreation facilities [1–5]	Total	1.99	-1.53, 5.52	.268	0.24	-2.20, 2.67	.850	4.71	-0.22, 9.63	.062
	Direct	1.85	-2.25, 5.95	.378	-0.17	-2.97, 2.63	.906	4.14	-1.49, 9.78	.150
Park proximity [1–5]	Total	2.40	-0.15, 4.95	.066	1.90	0.15, 3.66	.034	3.54	0.02, 7.06	.049
	Direct	3.40	0.54, 6.27	.020	3.07	1.07, 5.06	.003	3.12	-0.79, 7.03	.119
Accessibility & walking facilities [1–4]	Total	5.64	0.61, 10.66	.028	2.04	-1.38, 5.46	.242	11.31	4.40, 18.22	.001
	Direct	6.09	0.78, 11.39	.025	2.65	-0.93, 6.22	.147	11.45	4.06, 18.84	.002
*Adolescent-reported home environment*										
Electronic devices in the bedroom [0–6]	Total^a^	1.15	-0.49, 2.79	.170	1.46	0.34, 2.59	.011	-	-	-
**Associations in females**										
*Parent-reported Neighbourhood environment*										
Land use mix – diversity [1–5]	Total	-3.76	-6.99, -0.53	.023	-	-	-	-5.43	-10.27, -0.59	.028
	Direct	-3.17	-7.00, 0.67	.107	-	-	-	-5.81	-11.54, -0.08	.048
Transit stop proximity [1–5]	Total	-2.49	-4.97, -0.003	.049	-1.94	-3.67, -0.21	.028	-	-	-
	Direct	-2.08	-4.73, 0.57	.123	-1.47	-3.31, 0.37	.117	-	-	-
Recreation facilities [1–5]	Total	-4.99	-7.54, -1.24	.006	-3.24	-5.40, -1.09	.003	-1.93	-6.31, 2.45	.388
	Direct	-4.58	-8.28, -0.88	.015	-3.68	-6.19, -1.17	.004	-2.50	-7.71, 2.71	.347
Park proximity [1–5]	Total	-2.11	-4.42, 0.19	.072	-1.28	-2.86, 0.30	.113	-1.96	-5.14, 1.23	.228
	Direct	-1.30	-3.91, 1.31	.329	-0.23	-2.05, 1.58	.802	-2.57	-6.18, 1.03	.162
Accessibility & walking facilities [1–4]	Total	-6.74	-11.35, -2.12	.004	-5.22	-8.41, -2.02	.001	-6.24	-12.70, 0.21	.058
	Direct	-6.41	-11.32, -1.50	.011	-4.64	-8.01, 1.28	.007	-6.33	-13.24, 0.58	.073
*Adolescent-reported Home environment*										
Electronic devices in the bedroom [0–6]	Total^a^	-1.51	-3.27, 0.25	.093	-0.85	-2.05, 0.36	.169	-	-	-

*b,* regression coefficient; *CI* Confidence interval, *p p*-value,—not computed because the sex was not a moderator of the associations. ^a^Total and direct effects are equivalent as no mediating variables of characteristic-outcome associations were included in the models. All analyses were performed on 20 imputed datasets. Complete case analyses are in Tables S11, S13 and S15 (Supplementary Material 2). Model covariates are in Table S9 (Supplementary Material 2). Statistically significant effects (*p* < .05) are in bold

## Discussion

There were several notable findings from this study of 6,302 adolescents from 14 diverse countries. First, average total ST (based on accelerometer data) was substantial and varied across cities/countries, from 7.8 to 10.5 h/day. Second, having a personal social media account was associated with higher reported recreational screen time, accelerometer-based total ST, and ST during out-of-school periods on school days. Third, adolescents who reported less recreational screen time lived in neighbourhoods with more land use mix–diversity and had better perceptions of safety from traffic and crime than others. Fourth, girls who lived in neighbourhoods designed to support physical activity, such as with multiple recreation facilities, had less total ST on multiple accelerometer-based measures.

The World Health Organization (WHO) recommends no more than 2–3 h/day of ST for youth [7, 33]. Estimates of adolescent-reported recreational screen time in the present study varied from 2.4 h/day in Chennai, India to 5.5 h/day in Curitiba, Brazil. The average screen time exceeded 3 h/day for 14 of the 16 cities. These high levels of ST and recreational screen time are generally consistent with international studies of both HIC’s and Low Middle-Income Countries (LMIC’s), with 46% of adolescents exceeding 3 h per day of screen time across many countries and 73% in Columbia [34–36].

A caveat here is that the recent WHO guidelines document on physical activity and SB [37] also acknowledges that some sedentary activities can benefit cognitive function and social interaction in children and adolescents and that the negative health effects of SB is stronger for television viewing or recreational screen time than for total ST.

In our study, social media use emerged as one of the strongest potential risk factors for high total screen time that appears to contribute to more total ST. Adolescents with a personal social media account reported, on average, 37 more daily minutes of screen time than their counterparts, and the association between having a social media account and accelerometer-based ST was strongest for out-of-school periods on school days. There is increasing concern about negative mental health consequences of high social media use, supported by numerous studies [38, 39]. This warrants monitoring of social media use, screening of content by parents and limiting overall time spent on social media. Among recommendations to reduce adolescent social media use, the US Surgeon General advised actions for parents, policy makers, technology companies, and adolescents themselves [40]. He specifically spoke about bringing in warning labels on social medial platforms similar to those on cigarette packs advising parents that using these platforms can be harmful to adolescents’ physical and mental health. Technology companies could develop restricted usage norms for adolescents to help with not only usage analysis, but also the safety and security of adolescents.

In the present study, 4 of 5 cities in LMICs had significant positive associations between having a social media account and recreational screen time, compared to only 2 of 11 cities in HICs. A possible explanation is that adolescents in HICs have more access or opportunities to be engaged in other activities than do youth in LMICs. Another study found associations between SES and SB that were different in HIC’s and LMIC’s and varied by domain of SB [41]. Together, these findings suggest that different approaches may be required when developing intervention strategies to reduce SB in adolescents in different parts of the world. This pattern further points to a more urgent need for interventions to reduce adolescent use of social media in LMIC’s.

Both number of electronic devices in the bedroom and personal electronic devices were significantly associated with more recreational screen time, but not accelerometer-based ST. Other studies revealed differences in correlates of children’s screen time compared to overall ST. For example, the Healthy Active Preschool and Primary Years (HAPPY) study from Australia showed 8 yr. olds spent 99.6 and 119.3 min /day in screen time on weekdays and weekend days, respectively, compared to 119.3 and 374.6 min/day total sitting time on weekdays and weekends, respectively [14]. Correlates of recreational screen time in the present study are consistent with many prior studies [22, 42–46], but most earlier studies examined only electronic devices in the bedroom. Because portable devices, especially cell phones and music players, can be used while being active, this may explain why access to portable devices was not associated with more total device-measured ST in the present study.

Neighbourhood environment attributes were related to recreational screen time. Land use mix–diversity means there are multiple destinations within walking distance, so these opportunities may draw adolescents away from screens. Globally, road traffic crashes are a leading cause of death among young people, and the leading cause of death among 15–29-year-olds [47]. The largest effect size in the present study was for the negative association between traffic safety and screen time, possibly because traffic hazards are common injury risks worldwide. Present results support a hypothesis that better traffic safety and safety from crime may make both adolescents and parents more comfortable with teens spending more time in the neighbourhood, which in turn might reduce screen time.

Significant neighbourhood environment correlates of transport-related sitting were land use mix–diversity, proximity to recreation facilities, accessibility and walking facilities, and walking infrastructure and safety. More favourable scores on these variables were linked with less transport-related sitting. Present findings are generally consistent with literature indicating that in neighbourhoods designed for active transport, residents, including adolescents, can walk and cycle more for transport and are less dependent on automobiles [48–52]. Land use mix–diversity and recreation facilities are determined by land use policies that affect the layout of communities. The two variables related to design of walking facilities and streetscapes are determined by investments in streetscapes that create safe and attractive places for pedestrians. Thus, present results suggest zoning laws that favor mixed land use and investments in well-designed pedestrian infrastructure could reduce time sitting in cars, in addition to promoting active transportation [49].

More comprehensive measures of SB and analyses that evaluated moderation by city and sex likely contributed to the complexity of present findings. Unexpectedly, park proximity and accessibility and walking facilities were related to more ST on multiple measures, but only among boys. Though an explanation is not obvious, it is possible boys who could easily walk to parks and other destinations, perhaps including friends’ homes, were mainly sedentary when they arrived at their destinations. Additional research is needed to explain this surprising finding. For girls, there was substantial evidence that those who lived in neighbourhoods designed to support physical activity had less total ST on multiple accelerometer-based measures. The significant protective variables were land use mix-diversity, proximity to recreation facilities, and accessibility and walking facilities. The implication of these results is designing neighbourhoods to support physical activity may have particular benefits for reducing girls’ ST.

In general, the total and direct associations of parent-reported neighbourhood environment attributes with adolescent SB and ST outcomes were similar, indicating the potential effects of the neighbourhood environment on ST were not strongly mediated by access to personal electronic devices at home or having a social media account. Only a couple of neighbourhood attributes (transit stop proximity in females; park proximity in males) showed a change in associations with ST after accounting for the home environment. The neighbourhood and home environments appear to have mainly independent effects on adolescents’ SB and ST.

### Strengths and limitations

The large sample size from 14 countries with diversity in culture and environmental characteristics was a major strength. Other strengths were use of comparable methods of participant recruitment and data collection across study sites, stratified sampling ensuring participants were balanced by two important characteristics that impact physical activity (i.e., walkability and SES), examination of both home and neighbourhood environment measures, multiple measures of SB and ST outcomes, and use of validated measures.

The cross-sectional nature of the study is a limitation that precludes making causal interpretations. Present analyses were limited to reported environment attributes, but some of the attributes, especially in home environments, have no available objective measures. Subsequent analyses are planned to examine neighbourhood environment attributes using geographic information systems (GIS). It is a limitation that no lower-income countries participated. The electronic device and social media landscape continues to evolve, and it is difficult for research to reflect the constantly-changing media environment. The study used accelerometers to assess ST, but accelerometers do not distinguish between standing still and sitting, so measurement of ST was not optimal. Data were collected across different years for each country, all pre-pandemic, but the general consistency of results across countries supports confidence in the findings. In each country, we studied only one or two cities which may not be generalizable to the entire country or to rural areas. Though measures had evidence of test–retest reliability and construct validity, they were not validated in all participating countries. We accept that one of the limitations of the study is an implicit assumption that social media use is sedentary, but we recognize this may not always be so. We consider the associations of having a social media account with multiple measures of SB is an indication that social media use is often or usually a SB.

Results do not reflect any effects of the COVID-19 pandemic, but the documented increases in adolescent ST during the pandemic [3, 4, 53] make it even more important to understand influences on SB and ST, so as to inform intervention opportunities and priorities.

## Conclusions

The IPEN Adolescent results from 14 diverse countries show high prevalence of total sedentary time, recreational screen time, and transport-related sitting among adolescents. Despite differences in culture, built environments, and extent of sedentary time, patterns of association were generally similar across countries. Both home and neighbourhood environment attributes were related to multiple sedentary outcomes. A key finding was having a social media account was a strong driver of adolescent screen time and total sedentary time. Because social media use is also negatively related to adolescent mental health [37, 38], interventions to reduce access to, or regulate social media use by adolescents, should be developed and evaluated. Present results suggest more parent controls on access to personal electronic devices in the bedroom could yield health benefits for adolescents.

Perceptions of traffic safety and safety from crime appear to be important preconditions for adolescents to get out of their homes and away from screens. Activity-supportive neighbourhood environments may benefit girls more than boys, and further research is needed to identify reasons behind the sex differences.

## Supplementary Information

Supplementary Material 1: Supplementary file 1: Supplementary Table 1. Overall and site-specific sample characteristics (subsample with comparable accelerometer data, *N* = 3982). Supplementary Table 2. City-specific effects of perceived home environment characteristics on adolescents’ screen time. Supplementary Table 3. City-specific total effects of parent-perceived neighbourhood environment characteristics on adolescents’ transport-related sitting time. Supplementary file 2. All the Acyclic graphs and accompanying tables (15 of them).

## Data Availability

Data sharing is not currently available because multiple manuscripts examining IPEN adolescent data are in progress. IPEN study methods, surveys, protocols, and publications are available on the IPEN website: https://ipenproject.org/the-international-physical-activity-and-the-environment-network-about-us/ipen-studies/ipen-adolescent-study/.

## Abbreviations

SB: Sedentary Behaviour
COVID: Corona Virus
ST: Sedentary Time
IPEN: International Physical activity and the Environment Network
USA: United States of America
HIC: High Income Country
AUS: Australia
BEL: Belgium
CZE: Czech Republic
DNK: Denmark
CHN: China
ESP: Spain
ISR: Israel
PRT: Portugal
MYS: Malaysia
BRA: Brazil
IND: India
BGD: Bangladesh
NGA: Nigeria
AU: Administrative Units
SES: Socioeconomic Status
ID: Identity
LFE: Low Frequency Extension
TV: Television
DVD: Digital Versatile Disc
Min: Minutes
NEWS-Y: Neighbourhood Environment Walkability Scale for Youth
DAG: Directed Acyclic Graph
VIF: Variance Inflation Factor
GAMM: Generalised Additive Mixed Model
AIC: Akaike Information Criterion
Hour: Hr
WHO: World Health Organization
LMICs: Low Middle-Income Countries
HAPPY: Healthy Active Preschool and Primary Years
GIS: Geographic Information Systems
