# Supplementary material for: Associations of perceived neighbourhood and home environments with sedentary behaviour among adolescents in 14 countries: the IPEN adolescent cross sectional observational study
Source: Int J Behav Nutr Phys Act. 2024 Nov 29;21:136. doi: 10.1186/s12966-024-01678-4 (PMC11606016; doi:10.1186/s12966-024-01678-4)
Supplement: Supplementary file 1 — Supplementary Material 1: Supplementary file 1: Supplementary Table 1. Overall and site-specific sample characteristics (subsample with comparable accelerometer data, N = 3982). Supplementary Table 2. City-specific effects of perceived home environment characteristics on adolescents’ screen time. Supplementary Table 3. City-specific total effects of parent-perceived neighbourhood environment characteristics on adolescents’ transport-related sitting time. Supplementary file 2. All the Acyclic graphs and accompanying tables (15 of them). [file 12966_2024_1678_MOESM1_ESM.docx]

**Title: ASSOCIATIONS OF PERCEIVED NEIGHBOURHOOD AND HOME ENVIRONMENTS WITH SEDENTARY BEHAVIOUR AMONG ADOLESCENTS IN 14 COUNTRIES: IPEN ADOLESCENT STUDY**

**TABLE OF CONTENT**

| **Content** | **Page number** |
| --- | --- |
| **SUPPLEMENTARY FILE-1** Supplementary Table 1. Overall and site-specific sample characteristics (subsample with comparable accelerometer data, N=3982)- | 2-5 |
| Supplementary Table 2. City-specific effects of adolescent-reported home environment characteristics on adolescents’ screen time | 6 |
| Supplementary Table 3. City-specific total effects of parent-perceived neighbourhood environment characteristics on adolescents’ transport-related sitting time | 7-8 |
| **SUPPLEMENTARY FILE-2** Figure S1. Directed acyclic graph (DAG) depicting the hypothesised relations between characteristics of the home and neighbourhood environments with screen time. | 9 |
| Table S1. Covariates of regression models of perceived neighbourhood and home environment characteristics (exposures) and adolescents’ screen time (outcome) | 10-11 |
| Table S2. Total and direct effects of perceived neighbourhood and home environment characteristics on adolescents’ screen time [complete case analyses; N=4975] | 12 |
| Table S3. Adolescents’ sex as a moderator of total and direct effects of perceived neighbourhood and home environment characteristics on adolescents’ screen time | 13 |
| Table S4. City as a moderator of total effects of perceived home environment characteristics on adolescents’ screen time | 14 |
| Figure S2. Directed acyclic graph (DAG) depicting the hypothesised relations between characteristics of the neighbourhood environment with transport-related sitting time. | 15 |
| Table S5. Covariates of regression models of perceived neighbourhood environment characteristics (exposures) and adolescents’ transport-related sitting time (outcome) | 16 |
| Table S6. Total and direct effects of perceived neighbourhood environment characteristics on adolescents’ transport-related sitting time [complete case analyses; N=4975] | 17 |
| Table S7. Adolescents’ sex as a moderator of total and direct effects of perceived neighbourhood environment characteristics on adolescents’ transport-related sitting time | 18 |
| Table S8. City as a moderator of the total and direct effects of neighbourhood environment characteristics on adolescents’ transport-related sitting | 19-20 |
| Figure S3. Directed acyclic graph (DAG) depicting the hypothesised relations between characteristics of the neighbourhood and home environments with accelerometer-assessed sedentary time. | 21 |
| Table S9. Covariates of regression models of perceived neighbourhood and home environment characteristics (exposures) and adolescents’ accelerometer-assessed sedentary time (outcome) | 22-23 |
| Table S10. Total and direct effects of perceived neighbourhood and home environment characteristics on adolescents’ accelerometer-assessed total sedentary time | 24 |
| Table S11. Child’s sex as a moderator of total and direct effects of perceived neighbourhood and home environment characteristics on adolescents’ accelerometer-assessed total sedentary time | 25-26 |
| Table S12. Total and direct effects of perceived neighbourhood and home environment characteristics on adolescents’ accelerometer-assessed sedentary time during out-of-school periods on school days [complete case analyses; N=3148] | 27 |
| Table S13. Child’s sex as a moderator of total and direct effects of perceived neighbourhood and home environment characteristics on adolescents’ accelerometer-assessed to sedentary time during out-of-school periods on school days | 28-29 |
| Table S14. Total and direct effects of perceived neighbourhood and home environment characteristics on adolescents’ accelerometer-assessed sedentary time on non-school days [complete case analyses; N=3148] | 30 |
| Table S15. Child’s sex as a moderator of total and direct effects of perceived neighbourhood and home environment characteristics on adolescents’ accelerometer-assessed to sedentary time on non-school days | 31-32 |

**SUPPLEMENTARY FILE 1**

**Supplementary Table 1. Overall and site-specific sample characteristics (subsample with comparable accelerometer data, N=3982)**

|  |  | **High-income countries** | | | | | | | | | | | | **Low-middle-income countries** | | | | |  |  |  |  |  |
| --- | --- | --- | --- | --- | --- | --- | --- | --- | --- | --- | --- | --- | --- | --- | --- | --- | --- | --- | --- | --- | --- | --- | --- |
|  | **All sites** | **Melb**  **AUS** | **Ghent**  **BEL** | | **Hradec Kralove**  **CZE** | **Olomouc**  **CZE** | **Odense**  **DNK** | **Hong Kong**  **CHN** | **Haifa**  **ISR** | **Various cities**  **PRT** | **Valencia**  **ESP** | **Baltimore**  **USA** | **Seattle**  **USA** | **Dhaka**  **BGD** | **Curitiba**  **BRA** | **Chennai IND** | **Kuala Lumpur**  **MYS** | **Gombe**  **NGA** |  |  |  |  |  |
| **N** | 3982 | 372 | 224 | | 49 | 56 | 0 | 549 | 223 | 143 | 373 | 436 | 317 | 90 | 419 | 161 | 325 | 245 |  |  |  |  |  |
| ***Socio-demographics*** | | |  | |  |  |  |  |  |  |  |  |  |  |  |  |  |  |  |  |  |  |  |
| **Child’s age (years)** | |  |  | |  |  |  |  |  |  |  |  |  |  |  |  |  |  |  |  |  |  |  |
| Mean  (SD) | 14.6  (1.7) | 14.9  (1.6) | 13.3  (1.4) | | 15.8  (1.7) | 13.9  (2.0) | - | 14.4  (1.7) | 15.3  (1.4) | 15.9  (1.2) | 16.6  (0.8) | 14.1  (1.4) | 14.0  (1.4) | 13.9  (1.8) | 14.1  (1.7) | 13.8  (1.6) | 14.6  (1.2) | 15.3  (1.6) |  |  |  |  |  |
| **Child’s sex** |  |  |  | |  |  |  |  |  |  |  |  |  |  |  |  |  |  |  |  |  |  |  |
| % male | 45.7 | 38.7 | 39.7 | | 49.0 | 33.9 | - | 45.4 | 39.9 | 37.1 | 43.4 | 46.8 | 55.2 | 53.3 | 48.7 | 57.1 | 40.3 | 55.1 |  |  |  |  |  |
| **Highest education in household** | | |  | |  |  |  |  |  |  |  |  |  |  |  |  |  |  |  | |  |  |  |
| % College or higher  % missing | 51.8  7.7 | 35.5  46.0 | 75.5  1.8 | | 40.8  55.1 | 28.6  41.1 | - | 36.8  0.0 | 61.4  1.4 | 36.4  15.4 | 56.0  0.0 | 74.3  0.7 | 76.3  0.0 | 57.8  0.0 | 42.7  0.0 | 50.3  0.6 | 35.4  14.2 | 54.3  2.9 |  |  |  |  |  |
| **Area-level SES** | |  |  | |  |  |  |  |  |  |  |  |  |  |  |  |  |  |  |  |  |  |  |
| % High | 48.9 | 46.8 | 53.6 | | 69.4 | 73.2 | - | 46.6 | 51.6 | 54.6 | 52.0 | 51.4 | 48.3 | 44.4 | 43.9 | 50.9 | 46.5 | 41.2 |  |  |  |  |  |
| ***Adolescent-reported home environment*** | | |  | |  |  |  |  |  |  |  |  |  |  |  |  |  |  |  | |  |  |  |
| **Electronic devices in the bedroom [0-6]** | | | | |  |  |  |  |  |  |  |  |  |  |  |  |  |  |  | |  | |  |
| Mean  (SD)  % missing | 2.4  (1.6)  3.0 | 2.7  (1.5)  0.3 | 2.4  (1.5)  0.4 | | 2.8  (1.0)  0.0 | 2.6  (1.1)  0.0 | - | 1.8  (1.6)  0.5 | 3.0  (1.5)  0.0 | 3.3  (1.6)  0.1 | 2.7  (1.4)  0.0 | 2.7  (1.7)  0.0 | 2.4  (1.7)  0.0 | 2.0  (1.6)  0.0 | 2.6  (1.5)  0.0 | 1.2  (1.3)  1.3 | 1.8  (1.5)  0.4 | 2.0  (1.7)  0.0 |  |  |  |  |  |
| **Personal electronic devices [0-3]** | | | |  |  |  |  |  |  |  |  |  |  |  |  |  |  |  |  | |  |  |  |
| Mean  (SD)  % missing | 1.8  (0.9)  1.8 | 1.9  (0.8)  0.3 | 1.9  (0.7)  0.5 | | 1.8  (0.7)  0.0 | 1.5  (0.6)  0.0 | - | 1.8  (0.8)  0.5 | 1.6  (0.7)  0.0 | 2.1  (0.8)  0.1 | 2.3  (0.7)  0.0 | 2.2  (0.8)  0.0 | 2.3  (0.8)  0.0 | 1.0  (0.9)  0.0 | 1.3  (0.7)  0.0 | 0.6  (0.8)  0.0 | 1.5  (0.9)  0.4 | 1.1  (0.8)  0.0 |  |  |  |  |  |
| **Having own social media** | | |  | |  |  |  |  |  |  |  |  |  |  |  |  |  |  |  | |  |  |  |
| % Yes  % missing | 76.3  1.8 | 87.9  3.5 | 69.2  8.0 | | 95.9  0.0 | 87.5  0.0 | - | 87.8  3.6 | 86.1  0.0 | 89.5  2.8 | 80.7  0.0 | 70.0  0.0 | 66.3  0.0 | 28.9  1.1 | 92.6  0.0 | 26.7  0.0 | 74.2  4.9 | 60.0  0.0 |  |  |  |  |  |
| ***Parent-reported neighbourhood environment*** | | | | |  |  |  |  |  |  |  |  |  |  |  |  |  |  |  | |  | |  |
| **Residential density [0-1000]** | | |  | |  |  |  |  |  |  |  |  |  |  |  |  |  |  |  | |  |  |  |
| Mean  (SD)  % missing | 181.5  (200)  10.9 | 47.2  (104.4)  4.0 | 62.1  (91.7)  0.03 | | 121.1  (116.9)  0.6 | 134.6  (98.8)  0.5 | - | 449.2  (222)  0.0 | 214.4  (142)  0.03 | 125.8  (94.6)  0.8 | 246.1  (131.8)  0.0 | 39.6  (59.7)  1.5 | 21.8  (28.02)  0.7 | 177.3  (83.5)  0.0 | 95.4  (127.1)  0.0 | 66.1  (76.8)  0.0 | 280.5  (212.0)  2.5 | 260.9  (155.7)  0.2 |  |  |  |  |  |
| **Land use mix-diversity^1^ [1-5]** | | | | |  |  |  |  |  |  |  |  |  |  |  |  |  |  |  | |  | |  |
| Mean  (SD)  % missing | 3.2  (0.9)  8.3 | 3.0  (0.8)  4.0 | 3.4  (0.8)  0.1 | | 3.2  (0.8)  0.6 | 3.3  (0.9)  0.5 | - | 3.4  (0.8)  0.0 | 3.0  (0.7)  0.0 | 3.5  (0.8)  0.8 | 4.2  (0.5)  0.0 | 2.7  (0.9)  0.03 | 2.7  (0.9)  0.0 | 3.4  (0.7)  0.03 | 3.0  (0.7)  0.0 | 3.4  (0.7)  0.0 | 2.7  (0.7)  2.2 | 3.4  (0.8)  0.2 |  |  |  |  |  |
| **Recreation facilities^2^ [1-5]** | | |  | |  |  |  |  |  |  |  |  |  |  |  |  |  |  |  | |  |  |  |
| Mean  (SD)  % missing | 2.7  (0.9)  8.3 | 2.8  (0.8)  4.0 | 2.7  (0.8)  0.1 | | 3.3  (0.7)  0.6 | 3.1  (0.9)  0.5 | - | 2.8  (0.9)  0.0 | 2.4  (0.8)  0.0 | 2. 6  (0.8)  0.8 | 2.9  (0.8)  0.0 | 2.9  (0.9)  0.03 | 2.9  (0.8)  0.0 | 2.0  (0.7)  0.03 | 2.4  (0.8)  0.0 | 1.8  (0.6)  0.0 | 2.3  (0.9)  2.2 | 2.8  (0.5)  0.2 |  |  |  |  |  |
| **Accessibility and walking facilities [1-4]** | | | | |  |  |  |  |  |  |  |  |  |  |  |  |  |  |  | |  | |  |
| Mean  (SD)  % missing | 3.0  (0.6)  8.3 | 3.2  (0.5)  4.1 | 3.0  (0.6)  0.1 | | 3.3  (0.4)  0.6 | 3.3  (0.5)  0.5 | - | 3.0  (0.5)  0.0 | 3.1  (0.5)  0.03 | 3.0  (0.4)  0.7 | 3.6  (0.4)  0.0 | 3.0  (0.6)  0.03 | 2.8  (0.6)  0.0 | 2.8  (0.6)  0.0 | 2.9  (0.7)  0.0 | 2.6  (0.6)  0.0 | 2.7  (0.4)  2.1 | 2.8  (0.7)  0.2 |  |  |  |  |  |
| **Aesthetics [1-4]** | |  |  | |  |  |  |  |  |  |  |  |  |  |  |  |  |  |  |  |  |  |  |
| Mean  (SD)  % missing | 2.6  (0.8)  8.4 | 2.9  (0.8)  4.1 | 2.3  (0.7)  0.08 | | 2.4  (0.5)  0.6 | 2.2  (0.7)  0.5 | - | 2.6  (0.7)  0.0 | 2. 6  (0.8)  0.03 | 2.4  (0.5)  0.8 | 2.2  (0.8)  0.0 | 3.0  (0.7)  0.03 | 3.1  (0.7)  0.0 | 1.8  (0.8)  0.0 | 2.4  (0.9)  0.0 | 1.6  (0.9)  0.0 | 2.5  (0.6)  2.2 | 2.9  (0.9)  0.18 |  |  |  |  |  |
| **Traffic safety [1-4]** | |  |  | |  |  |  |  |  |  |  |  |  |  |  |  |  |  |  |  |  |  |  |
| Mean  (SD)  % missing | 2.6  (0.7)  8.2 | 2.9  (0.6)  4.1 | 2.5  (0.6)  0.1 | | 3.2  (0.4)  0.6 | 2.9  (0.6)  0.5 | - | 2.9  (0.5)  0.0 | 2.4  (0.7)  0.0 | 2.8  (0.5)  0.7 | 2.6  (0.7)  0.0 | 2.5  (0.6)  0.03 | 2.7  (0.6)  0.0 | 2.4  (06)  0.0 | 2.2  (0.8)  0.0 | 2.3  (0.7)  0.0 | 2.4  (0.5)  2.1 | 3.0  (0.9)  0.2 |  |  |  |  |  |
| **Pedestrian infrastructure & safety [1-4]** | | | | |  |  |  |  |  |  |  |  |  |  |  |  |  |  |  | |  | |  |
| Mean  (SD)  % missing | 2.8  (0.7)  8.2 | 2.8  (0.6)  4.1 | 2.7  (0.6)  0.1 | | 2. 9  (0. 8)  0.6 | 2.9  (0.5)  0.5 | - | 3.0  (0.5)  0.0 | 2.9  (0.8)  0.0 | 2.9  (0.5)  0.7 | 3.0  (0.6)  0.0 | 2.8  (0.7)  0.03 | 2.9  (0.6)  0.0 | 2.5  (0.6)  0.0 | 2.6  (0.8)  0.0 | 2.9  (0.8)  0.0 | 2.7  (0.6)  2.1 | 3.0  (0.8)  0.2 |  |  |  |  |  |
| **Crime safety [1-4]** | | |  | |  |  |  |  |  |  |  |  |  |  |  |  |  |  |  | |  |  |  |
| Mean  (SD)  % missing | 2.8  (0.9)  8.3 | 3.1  (0.8)  4.1 | 3.1  (0.7)  0.1 | | 3.0  (0.8)  0.6 | 2.7  (0.7)  0.5 | - | 2.7  (0.9)  0.0 | 3.3  (0.9)  0.1 | 3.0  (0.6)  0.7 | 3.2  (0.8)  0.0 | 3.0  (0.7)  0.03 | 3.1  (0.7)  0.0 | 2.0  (0.9)  0.0 | 2.1  (0.8)  0.0 | 3.0  (1.1)  0.0 | 2.1  (0.7)  2.1 | 2.7  (1.2)  0.2 |  |  |  |  |  |
| **Transit stop proximity [1-5]** | | |  | |  |  |  |  |  |  |  |  |  |  |  |  |  |  |  | |  |  |  |
| Mean  (SD)  % missing | 4.1  (1.2)  8.8 | 4.5  (0.8)  4.0 | 4.7  (0.7)  0.3 | | 4.4  (1.1)  0.6 | 4.3  (0.9)  0.5 | - | 3.9  (1.1)  0.0 | 4.5  (0.9)  0.1 | 4.5  (0.8)  0.8 | 4.8  (0.6)  0.0 | 3.7  (1.4)  0.03 | 4.0  (1.2)  0.0 | 2.2  (1.3)  0.1 | 4.8  (0.6)  0.0 | 3.9  (1.1)  0.03 | 3.3  (1.3)  2.2 | 4.0  (1.0)  0.3 |  |  |  |  |  |
| **Park proximity [1-5]** | |  |  | |  |  |  |  |  |  |  |  |  |  |  |  |  |  |  |  |  |  |  |
| Mean  (SD)  % missing | 3.0  (1.3)  8.8 | 3.8  (1.0)  4.0 | 3.2  (1.3)  0.1 | | 3.3  (1.1)  0.6 | 2.8  (1.1)  0.5 | - | 3.4  (1.1)  0.0 | 3.2  (1.2)  0.0 | 2.9  (1.0)  0. 8 | 4.0  (0.8)  0.0 | 2.9  (1.2)  0.03 | 3.1  (1.1)  0.0 | 1.8  (0.9)  0.1 | 2.7  (0.9)  0.0 | 2.2  (1.1)  0.0 | 2.5  (1.1)  2.6 | 1.0  (0.0)  0.0 |  |  |  |  |  |
| ***Accelerometer-assessed sedentary time*** | | | | |  |  |  |  |  |  |  |  |  |  |  |  |  |  |  | |  | |  |
| **Total sedentary time (min/day)** | | |  | |  |  |  |  |  |  |  |  |  |  |  |  |  |  |  | |  |  |  |
| Mean  (SD) | 534 (89.2) | 506  (78.8) | 504  (76.0) | | 537  (95.5) | 470  (100) | - | 544  (80.4) | 520  (76.2) | 521  (63.7) | 632  (69.5) | 536  (82.7) | 524 (76.0) | 518  (98.4) | 509  (93.7) | 508  (82.9) | 563  (89.6) | 504  (78.9) |  |  |  |  |  |
| % weartime | 66.1 | 64.4 | 63.2 | | 66.7 | 59.1 |  | 70.1 | 64.7 | 65.5 | 73.5 | 65.8 | 65.6 | 61.4 | 63.1 | 62.0 | 68.6 | 60.8 |  |  |  |  |  |
| **Sedentary time during out-of-school periods on school days (min/day)** | | | | | | | | | | | | | | | | | | |  |  |  |  |  |
| Mean  (SD)  % missing | 302 (83.4)  0.03 | 280  (66.5)  0.0 | 260  (66.0)  0.0 | | 294  (80.9)  0.0 | 266  (79.7)  0.0 | - | 248  (73.2)  0.0 | 301 (67.6) 0.0 | 249  (73.7)  0.0 | 361  (62.1)  0.0 | 316  (72.2)  0.0 | 310  (66.3)  0.03 | 369 (84.1)  0.0 | 347  (95.3)  0.0 | 243 (60.9)  0.0 | 352 (69.8)  0.0 | 282  (69.4)  0.0 |  |  |  |  |  |
| % weartime | 63.8 | 63.3 | 61.3 | | 63.2 | 55.6 |  | 67.6 | 63.4 | 61.9 | 69.4 | 63.8 | 63.8 | 61.6 | 62.1 | 59.4 | 67.6 | 56.7 |  |  |  |  |  |
| **Sedentary time on non-school days (min/day)** | | | | | | |  |  |  |  |  |  |  |  |  |  |  |  |  |  |  |  |  |
| Mean  (SD)  % missing | 485  (108)  7.2 | 464  (108.8)  1.4 | 452  (91.1)  0.6 | | 494  (107.1)  0.0 | 429  (116.9)  0.3 | - | 500  (105)  0.1 | 476  (107)  1.1 | 489  (84.8)  0.6 | 539  (104.0)  0.1 | 475  (100.2)  0.8 | 481  (99.2)  0.5 | 482  (117)  0.9 | 461  (112.6)  0.7 | 475  (99.7)  0.1 | 521  (105.4)  0.7 | 468  (113.4)  0.5 |  |  |  |  |  |
| % weartime | 65.5 | 63.9 | 65.2 | | 64.5 | 60.8 |  | 69.7 | 67.3 | 68.1 | 72.5 | 63.8 | 65.5 | 59.7 | 62.9 | 60.9 | 67.9 | 57.8 |  |  |  |  |  |
| ***Accelerometer wear time information*** | | | | | |  |  |  |  |  |  |  |  |  |  |  |  |  |  |  |  |  |  |
| **Total valid days** | | |  | |  |  |  |  |  |  |  |  |  |  |  |  |  |  |  |  |  |  |  |
| Mean  (SD) | 6.9  1.4 | 6.9  (1.2) | 7.2  (1.4) | | 6.5  (1.2) | 6.2  (1.3) | - | 7.0  (1.8) | 7.2  (0.9) | 6.9  (1.1) | 6.3  (1.3) | 7.2  (1.4) | 7.3  (1.6) | 7.1  (0.7) | 6.7  (1.1) | 7.1  (1.0) | 7.0  (1.3) | 6.9  (1.1) |  |  |  |  |  |
| **Valid school days only** | | |  | |  |  |  |  |  |  |  |  |  |  |  |  |  |  |  |  |  |  |  |
| Mean  (SD)  % missing | 5.3  (1.2)  0.03 | 5.4  (0.8)  0.0 | 5.5  (1.1)  0.0 | | 4.2  (1.0)  0.0 | 4.6  (1.0)  0.0 | - | 5.3  (1.5)  0.0 | 6.4  (0.9)  0.0 | 5.1  (0.9)  0.0 | 4.3  (1.0)  0.0 | 5.5  (1.3)  0.0 | 5.5  (1.5)  0.03 | 5.7  (0.8)  0.0 | 5.0  (1.0)  0.0 | 5.4  (0.9)  0.0 | 5.4  (1.0)  0.0 | 5.3  (0.9)  0.0 |  |  |  |  |  |
| **Valid non-school days only** | | | | |  |  |  |  |  |  |  |  |  |  |  |  |  |  |  |  |  |  |  |
| Mean  (SD)  % missing | 1.8  (0.7)  7.2 | 1.7  (0.5)  1.4 | 1.9  (0.7)  0.6 | | 2.4  (0.8)  0.0 | 1.9  (0.4)  0.3 | - | 1.7  (0.7)  0.1 | 1.0  (0.2)  1.1 | 2.0  (0.5)  0.3 | 2.0  (0.6)  0.1 | 1.9  (0.7)  0.8 | 2.0  (0.7)  0.5 | 1.5  (0.7)  0.1 | 1.8  (0.6)  0.7 | 1.8  (0.7)  0.1 | 1.7  (0.5)  0.7 | 1.8  (0.6)  0.5 |  |  |  |  |  |
| **Average wear time minutes per valid days** | | | | | |  |  |  |  |  |  |  |  |  |  |  |  |  |  |  |  |  |  |
| Mean  (SD) | 808  (80.8) | 786  (74.1) | 797  (71.2) | | 805  (92.9) | 795  (79.9) | - | 776  (79.0) | 804  (67.0) | 796  (63.0) | 860  (63.3) | 814  (76.3) | 799  (71.4) | 844  (73.4) | 807  (85.1) | 819  (70.9) | 821  (95.0) | 829  (92.2) |  |  |  |  |  |
| **Average wear time minutes in non-school period on school days** | | | | | | | |  |  |  |  |  |  |  |  |  |  |  |  |  |  |  |  |
| Mean  (SD)  % missing | 473  (108)  0.03 | 442  (75.8)  0.0 | 424  (83.9)  0.0 | | 465  (104.3)  0.0 | 478  (99.0)  0.0 | - | 367  (93.0)  0.0 | 474  (90.5)  0.0 | 402  (98.9)  0.0 | 520  (67.5)  0.0 | 495  (80.7)  0.0 | 486  (77.1)  0.03 | 599  (79.2)  0.0 | 559  (127.0)  0.0 | 409  (84.2)  0.0 | 521  (76.0)  0.0 | 497  (100.7)  0.0 |  |  |  |  |  |
| **Average wear time minutes per non-school day** | | | | | | |  |  |  |  |  |  |  |  |  |  |  |  |  |  |  |  |  |
| Mean  (SD)  % missing | 740  (111)  7.2 | 726  (108.1)  1.4 | 709  (90.2)  0.6 | | 766  (97.6)  0.0 | 706  (94.0)  0.3 | - | 717  (108)  0.1 | 707  (109)  1.1 | 718  (84.7)  0.3 | 743  (109.2)  0.1 | 744  (107.2)  0.8 | 734  (103.4)  0.5 | 807  (103)  0.1 | 733  (107.9)  0.7 | 780  (103)  0.1 | 767  (119.8)  0.7 | 809  (127.4)  0.5 |  |  |  |  |  |

*Notes.* Melb=Melbourne; SES=Socio-economic-status; SD=standard deviation; min=minutes; AUS, Australia; BEL, Belgium; CZE, Czech Republic; DNK, Denmark; HKG, Hong Kong; CHN, China; ISR, Israel; PRT, Portugal; ESP, Spain; USA, Unites States of America; BGD, Bangladesh; BRA, Brazil; IND, India; MYS, Malaysia; NGA, Nigeria ^1^ excluding transit stops; ^2^ excluding parks

**Supplementary Table 2. City-specific effects of adolescent-reported home environment characteristics on adolescents’ screen time**

|  | Electronic devices in the bedroom | | | Having own social media | | |
| --- | --- | --- | --- | --- | --- | --- |
| City (country) | ***b*** | **95% CI** | ***p*** | ***b*** | **95% CI** | ***p*** |
| *High-income countries* |  |  |  |  |  |  |
| Melbourne (AUS) | **11.67** | **1.95, 21.38** | **.019** | **63.81** | **14.61, 113.02** | **.011** |
| Ghent (BEL) | **23.33** | **11.49, 35.17** | **<.001** | 8.60 | -31.02, 48.22 | .670 |
| Hradec Králové (CZE) | 11.48 | -4.58, 27.54 | .161 | 32.27 | -46.47, 111.00 | .422 |
| Olomouc (CZE) | **30.41** | **15.83, 45.00** | **<.001** | 64.68 | -3.55, 1232.92 | .063 |
| Odense (DNK) | **25.89** | **11.94, 39.84** | **<.001** | 28.91 | -19.18, 77.00 | .239 |
| Hong Kong (HKG, CHN) | **11.51** | **6.23, 16.78** | **<.001** | 5.29 | -23.68, 34.27 | .720 |
| Haifa (ISR) | 7.31 | -4.82, 19.44 | .238 | 40.24 | -14.46, 94.95 | .149 |
| Various cities (PRT) | 1.49 | -11.77, 14.76 | .825 | 17.28 | -60.27, 98.84 | .662 |
| Valencia (ESP) | 5.77 | -3.06, 14.61 | .201 | 22.90 | -11.76, 57.56 | .195 |
| Baltimore (USA) | **23.67** | **16.25, 31.08** | **<.001** | **53.79** | **25.03, 82.56** | **<.001** |
| Seattle (USA) | **16.92** | **8.72, 25.12** | **<.001** | 1.32 | -27.79, 30.43 | .929 |
| *Low-middle-income countries* |  |  |  |  |  |  |
| Dhaka (BGD) | **34.99** | **17.23, 52.75** | **<.001** | **95.87** | **31.61, 160.14** | **<.001** |
| Curitiba (BRA) | **14.90** | **6.82, 22.98** | **<.001** | **101.63** | **52.09, 151.16** | **<.001** |
| Chennai (IND) | 9.97 | -4.13, 24.07 | .167 | **51.02** | **15.07, 86.98** | **.005** |
| Kuala Lumpur (MYS) | **16.01** | **9.53, 22.49** | **<.001** | 14.29 | -10.85, 39.43 | .265 |
| Gombe (NGA) | **34.19** | **23.84, 44.55** | **<.001** | **116.90** | **81.37, 152.42** | **<.001** |

*Note.* *b,* regression coefficient; CI, confidence interval; *p*, p-value; AUS, Australia; BEL, Belgium; CZE, Czech Republic; DNK, Denmark; HKG, Hong Kong; CHN, China; ISR, Israel; PRT, Portugal; ESP, Spain; USA, Unites States of America; BGD, Bangladesh; BRA, Brazil; IND, India; MYS, Malaysia; NGA, Nigeria. Total and direct effects are equivalent as no mediating variables of characteristic-outcome associations were included in the models. All analyses were performed on 20 imputed datasets. Complete case analyses are in the Supplementary Material 2 (Tables S4). Model covariates are reported in Supplementary Material 2, Table S1. Statistically significant effects (*p*<.05) are in bold.

**Supplementary Table 3. City-specific total effects of parent-perceived neighbourhood environment characteristics on adolescents’ transport-related sitting time**

|  | **Land use mix diversity^1^** | | | **Accessibility and walking facilities** | | | | T**raffic safety** | | | |
| --- | --- | --- | --- | --- | --- | --- | --- | --- | --- | --- | --- |
| **City (country)** | ***e^b^*** | **95% CI** | *p* | ***e^b^*** | **95% CI** | *p* | | ***e^b^*** | **95% CI** | | *p* |
| *High-income countries* |  |  |  |  |  |  | |  |  |  | |
| Melbourne (AUS) | 0.85 | 0.72, 1.00 | .055 | 0.91 | 0.70, 1.20 | .517 | | 0.89 | 0.73, 1.09 | .267 | |
| Ghent (BEL) | **0.82** | **0.67, 1.00** | **.046** | 0.85 | 0.65, 1.11 | .237 | | 0.83 | 0.63, 1.09 | .175 | |
| Hradec Králové (CZE) | 0.94 | 0.73, 1.22 | .650 | 0.84 | 0.54, 1.31 | .444 | | 1.06 | 0.74, 1.53 | .736 | |
| Olomouc (CZE) | 1.00 | 0.77, 1.29 | .973 | 0.94 | 0.61, 1.45 | .791 | | 1.09 | 0.77, 1.53 | .624 | |
| Odense (DNK) | 0.85 | 0.70, 1.03 | .102 | **0.65** | **0.44, 0.96** | **.031** | | 1.24 | 0.98, 1.58 | .074 | |
| Hong Kong (HKG, CHN) | **0.88** | **0.80, 0.96** | **.003** | **0.74** | **0.65, 0.85** | **<.001** | | **0.88** | **0.77, 0.99** | **.047** | |
| Haifa (ISR) | 0.89 | 0.73, 1.07 | .218 | 1.05 | 0.77, 1.42 | .769 | | 1.08 | 0.86, 1.35 | .508 | |
| Various cities (PRT) | 0.82 | 0.65, 1.04 | .105 | 0.80 | 0.52, 1.25 | .335 | | 0.96 | 0.65, 1.43 | .858 | |
| Valencia (ESP) | **0.71** | **0.57, 0.88** | **.002** | 0.91 | 0.70, 1.18 | .471 | | 0.97 | 0.84, 1.13 | .708 | |
| Baltimore (USA) | 0.95 | 0.84, 1.07 | .381 | 0.95 | 0.79, 1.15 | .609 | | 1.05 | 0.87, 1.26 | .620 | |
| Seattle (USA) | 0.97 | 0.85, 1.11 | .648 | 0.92 | 0.77, 1.10 | .364 | | 0.98 | 0.80, 1.19 | .816 | |
| *Low-middle-income countries* |  |  |  |  |  |  | |  |  |  | |
| Dhaka (BGD) | **0.66** | **0.45, 0.98** | **.037** | 1.11 | 0.73, 1.70 | .611 | | 1.26 | 0.84, 1.89 | .266 | |
| Curitiba (BRA) | 0.97 | 0.82, 1.14 | .675 | 0.90 | 0.76, 1.06 | .233 | | 1.02 | 0.88, 1.17 | .801 | |
| Chennai (IND) | **0.71** | **0.58, 0.86** | **<.001** | 1.01 | 0.81, 1.27 | .902 | | 1.03 | 0.84, 1.26 | .781 | |
| Kuala Lumpur (MYS) | 0.94 | 0.83, 1.06 | .303 | 1.07 | 0.85, 1.34 | .580 | | 0.99 | 0.84, 1.17 | .924 | |
| Gombe (NGA) | 1.10 | 0.91, 1.33 | .337 | 1.11 | 0.88, 1.39 | .379 | | 0.85 | 0.71, 1.00 | .055 | |
|  | | | | | | | | | | | |
|  | **Pedestrian infrastructure & safety** | | | **Aesthetics** | | | | **Park proximity** | | | |
| **City (country)** | ***e^b^*** | **95% CI** | *p* | ***e^b^*** | **95% CI** | | *p* | ***e^b^*** | **95% CI** | | *p* |
| *High-income countries* |  |  |  |  |  | |  |  |  | |  |
| Melbourne (AUS) | 0.96 | 0.74, 1.25 | .764 | 0.93 | 0.77, 1.11 | | .430 | 0.97 | 0.86, 1.09 | | .591 |
| Ghent (BEL) | 0.90 | 0.69, 1.17 | .430 | 0.81 | 0.64, 1.02 | | .070 | 0.96 | 0.84, 1.10 | | .567 |
| Hradec Králové (CZE) | 0.93 | 0.65, 1.32 | .682 | 1.05 | 0.72, 1.53 | | .797 | 0.97 | 0.79, 1.19 | | .752 |
| Olomouc (CZE) | 0.97 | 0.67, 1.39 | .852 | 1.08 | 0.78, 1.50 | | .646 | 1.12 | 0.95, 1.33 | | .177 |
| Odense (DNK) | **0.78** | **0.61, 0.99** | **.044** | 0.83 | 0.65, 1.06 | | .136 | 0.90 | 0.78, 1.04 | | .167 |
| Hong Kong (HKG, CHN) | **0.82** | **0.73, 0.92** | **.001** | 1.00 | 0.91, 1.10 | | .999 | 1.01 | 0.95, 1.08 | | .770 |
| Haifa (ISR) | 0.96 | 0.78, 1.19 | .721 | 1.02 | 0.84, 1.23 | | .876 | 0.91 | 0.79, 1.04 | | .177 |
| Various cities (PRT) | 0.91 | 0.62, 1.32 | .614 | 0.97 | 0.70, 1.35 | | .866 | 1.02 | 0.86, 1.22 | | .819 |
| Valencia (ESP) | **0.80** | **0.67, 0.96** | **.014** | 0.88 | 0.75, 1.02 | | .086 | 1.10 | 0.96, 1.25 | | .179 |
| Baltimore (USA) | 0.92 | 0.78, 1.18 | .865 | 1.04 | 0.89, 1.21 | | .623 | 1.03 | 0.94, 1.13 | | .568 |
| Seattle (USA) | 0.98 | 0.82, 1.18 | .865 | 1.09 | 0.92, 1.30 | | .311 | 1.01 | 0.91, 1.12 | | .883 |
| *Low-middle-income countries* |  |  |  |  |  | |  |  |  | |  |
| Dhaka (BGD) | 0.75 | 0.49, 1.14 | .181 | 1.01 | 0.73, 1.38 | | .961 | 0.90 | 0.68, 1.19 | | .463 |
| Curitiba (BRA) | 0.88 | 0.77, 1.01 | .062 | 1.00 | 0.88, 1.14 | | .963 | 1.01 | 0.90, 1.13 | | .842 |
| Chennai (IND) | **1.36** | **1.15, 1.61** | **<.001** | **1.23** | **1.05, 1.45** | | **.013** | 0.99 | 0.87, 1.12 | | .877 |
| Kuala Lumpur (MYS) | 1.04 | 0.87, 1.24 | .668 | 1.03 | 0.87, 1.22 | | .739 | 1.02 | 0.94, 1.11 | | .629 |
| Gombe (NGA) | **1.21** | **1.00, 1.46** | **.047** | **1.20** | **1.00, 1.44** | | **.048** | 0.78 | 0.11, 5.57 | | .807 |

*Note.* *e^b^,* exponentiated regression coefficient; CI, confidence interval; *p*, p-value; AUS, Australia; BEL, Belgium; CZE, Czech Republic; DNK, Denmark; HKG, Hong Kong; CHN, China; ISR, Israel; PRT, Portugal; ESP, Spain; USA, Unites States of America; BGD, Bangladesh; BRA, Brazil; IND, India; MYS, Malaysia; NGA, Nigeria. The estimates above are estimates of total and direct effects for all environmental characteristics except for land use mix – diversity for which they represent total effects only. All analyses were performed on 20 imputed datasets. Complete case analyses are in the Supplementary Material (Tables S8, Supplementary Material 2). Model covariates are reported in Table S5 (Supplementary Material 2). Statistically significant effects (*p*<.05) are in bold.

**SUPPLEMENTARY FILE 2**

**
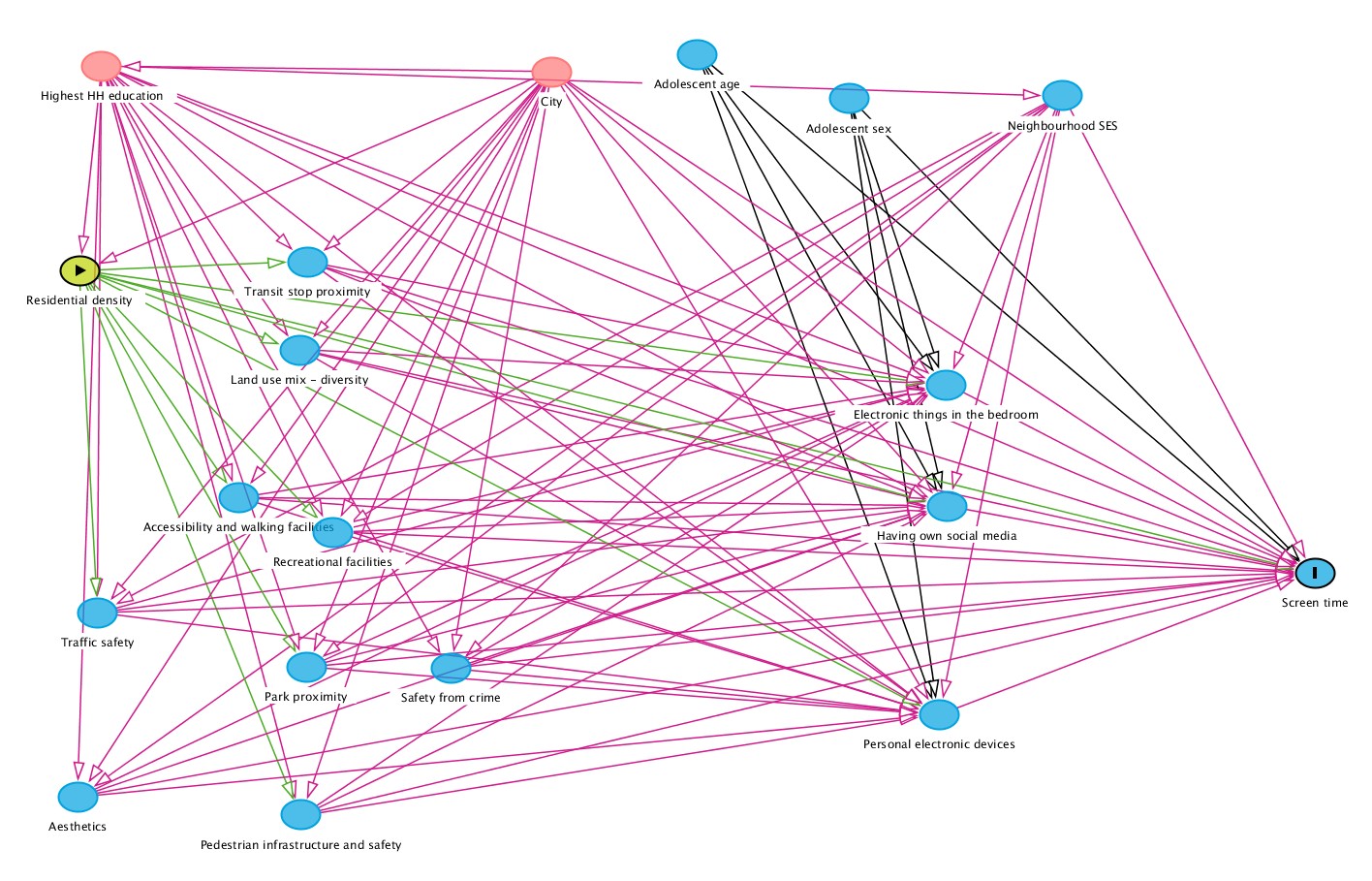
**

**Figure S1.** Directed acyclic graph (DAG) depicting the hypothesised relations between characteristics of the home and neighbourhood environments with screen time. Through the DAG, we identified which covariates to include in the statistical analyses to sufficiently control for potential confounders. This particular DAG was used to inform the model of the total effect of parental perceptions of neighbourhood residential density on adolescents’ screen time. Variables with red circles denote the set of potential confounders. A minimal sufficient set of confounders (included in the regression models) is a subset of this set of variables.

**Table S1. Covariates of regression models of perceived neighbourhood and home environment characteristics (exposures) and adolescents’ screen time (outcome)**

| **Model** | **Effect estimated** | **Covariates** |
| --- | --- | --- |
| 1T | Total effects on Residential density | adolescent age; adolescent sex; area-level SES; highest education in household; city |
| 1D | Direct effects of Residential density | *accessibility and walking facilities*; adolescent age; adolescent sex; aesthetics; city; *electronic devices in the bedroom*; *having own social media*; highest education in household; *land use mix – diversity*^1^; area-level SES; *pedestrian infrastructure and safety*; *personal electronic devices*; *recreation facilities*^2^; crime safety; *traffic safety*; *transit stop proximity*; *park proximity* |
| 2T | Total effects of Land use mix diversity^1^ | adolescent age; adolescent sex; area-level SES; highest education in household; city; residential density |
| 2D | Direct effects of Land use mix diversity^1^ | accessibility and walking facilities; adolescent age; adolescent sex; aesthetics; city; *electronic devices in the bedroom*; *having own social media*; highest education in household; residential density; area-level SES; pedestrian infrastructure and safety; *personal electronic devices*; recreation facilities^2^; crime safety; traffic safety; transit stop proximity; park proximity |
| 3T | Total effects of Transit stop proximity | adolescent age; adolescent sex; area-level SES; highest education in household; city; residential density |
| 3D | Direct effects of Transit stop proximity | accessibility and walking facilities; adolescent age; adolescent sex; aesthetics; city; *electronic devices in the bedroom*; *having own social media*; highest education in household; residential density; area-level SES; pedestrian infrastructure and safety; *personal electronic devices*; recreation facilities^2^; crime safety; traffic safety; park proximity |
| 4T | Total effects of Recreation facilities^2^ | adolescent age; adolescent sex; area-level SES; highest education in household; city; residential density |
| 4D | Direct effects of Recreation facilities^2^ | accessibility and walking facilities; adolescent age; adolescent sex; aesthetics; city; *electronic devices in the bedroom*; *having own social media*; highest education in household; land use mix - diversity^1^; area-level SES; pedestrian infrastructure and safety; *personal electronic devices*; residential density; crime safety; traffic safety; transit stop proximity; park proximity |
| 5T | Total effects of Park proximity | adolescent age; adolescent sex; area-level SES; highest education in household; city; residential density |
| 5D | Direct effects of Park proximity | accessibility and walking facilities; adolescent age; adolescent sex; aesthetics; city; *electronic devices in the bedroom*; *having own social media*; highest education in household; land use mix - diversity^1^; area-level SES; pedestrian infrastructure and safety; *personal electronic devices*; recreation facilities; residential density; crime safety; traffic safety; transit stop proximity |
| 6T | Total effects of Accessibility and walking facilities | adolescent age; adolescent sex; area-level SES; highest education in household; city; residential density |
| 6D | Direct effects of Accessibility and walking facilities | residential density; adolescent age; adolescent sex; aesthetics; city; *electronic devices in the bedroom*; *having own social media*; highest education in household; land use mix - diversity^1^; area-level SES; pedestrian infrastructure and safety; *personal electronic devices*; recreation facilities^2^; crime safety; traffic safety; transit stop proximity; park proximity |
| 7T | Total effects of Traffic safety | adolescent age; adolescent sex; area-level SES; highest education in household; city; residential density |
| 7D | Direct effects of Traffic safety | accessibility and walking facilities; adolescent age; adolescent sex; aesthetics; city; *electronic devices in the bedroom*; *having own social media*; highest education in household; land use mix - diversity^1^; area-level SES; pedestrian infrastructure and safety; *personal electronic devices*; recreation facilities^2^; crime safety; residential density; transit stop proximity; park proximity |
| 8T | Total effects of Pedestrians infrastructure and safety | adolescent age; adolescent sex; area-level SES; highest education in household; city; residential density |
| 8D | Direct effects of Pedestrians infrastructure and safety | accessibility and walking facilities; adolescent age; adolescent sex; aesthetics; city; *electronic devices in the bedroom*; *having own social media*; highest education in household; land use mix - diversity^1^; area-level SES; residential density; *personal electronic devices*; recreation facilities^2^; crime safety; traffic safety; transit stop proximity; park proximity |
| 9T | Total effects of Crime safety | adolescent age, adolescent sex, area-level SES; Highest education in household, city |
| 9D | Direct effects of Crime safety | accessibility and walking facilities; adolescent age; adolescent sex; aesthetics; city; *electronic devices in the bedroom*; *having own social media*; highest education in household; land use mix - diversity^1^; area-level SES, *pedestrian infrastructure and safety*; personal electronic devices; recreation facilities^2^; residential density; traffic safety; transit stop proximity; park proximity |
| 10T | Total effects of Aesthetics | adolescent age; adolescent sex; area-level SES; highest education in household; city |
| 10D | Direct effects of Aesthetics | accessibility and walking facilities; adolescent age; adolescent sex; residential density; city; *electronic devices in the bedroom*; *having own social media*; highest education in household; land use mix - diversity^1^; area-level SES; pedestrian infrastructure and safety; *personal electronic devices*; recreation facilities^2^; crime safety; traffic safety; transit stop proximity; park proximity |
| 11T | Total effects of personal electronic devices | accessibility and walking facilities; adolescent age; adolescent sex; aesthetics; city; electronic devices in the bedroom; having own social media; highest education in household; land use mix - diversity^1^; area-level SES; pedestrian infrastructure and safety; residential density; recreation facilities^2^; crime safety; traffic safety; transit stop proximity; park proximity |
| 11D | Direct effects of personal electronic devices | accessibility and walking facilities; adolescent age; adolescent sex; aesthetics; city; electronic devices in the bedroom; having own social media; highest education in household; land use mix – diversity^1^; area-level SES; pedestrian infrastructure and safety; residential density; recreation facilities^2^; crime safety; traffic safety; transit stop proximity; park proximity |
| 12T | Total effects of having own social media | accessibility and walking facilities; adolescent age; adolescent sex; aesthetics; city; highest education in household; land use mix - diversity^1^; area-level SES; pedestrian infrastructure and safety; recreation facilities^2^; residential density; crime safety; traffic safety; transit stop proximity; park proximity |
| 12D | Direct effects of having own social media | accessibility and walking facilities; adolescent age; adolescent sex; aesthetics; city; electronic devices in the bedroom; residential density; highest education in household; land use mix - diversity^1^; area-level SES; pedestrian infrastructure and safety; personal electronic devices; recreation facilities^2^; crime safety; traffic safety; transit stop proximity; park proximity |
| 13T | Total effects of electronic devices in the bedroom | accessibility and walking facilities; adolescent age; adolescent sex; aesthetics; city; highest education in household; land use mix - diversity^1^; area-level SES; pedestrian infrastructure and safety; recreation facilities^2^; residential density; crime safety; traffic safety; transit stop proximity; park proximity |
| 13D | Direct effects of electronic thing in the bedroom | accessibility and walking facilities; adolescent age; adolescent sex; aesthetics; city; residential density; having own social media; highest education in household; land use mix – diversity^1^; area-level SES; pedestrian infrastructure and safety; personal electronic devices; recreation facilities^2^; crime safety; traffic safety; transit stop proximity; park proximity |

*Note.* ^1^ excluding transit stops; ^2^ excluding parks; SES, socio-economic status. Potential mediators *in italics.*

**Table S2. Total and direct effects of perceived neighbourhood and home environment characteristics on adolescents’ screen time [complete case analyses; N=4975]**

| **Model** | **Effect estimated** | ***b*** | **95% CI** | ***p*-value** |
| --- | --- | --- | --- | --- |
| 1T | Total effects of Residential density | -0.01 | -0.04, 0.02 | 0.518 |
| 1D | Direct effects of Residential density | -0.01 | -0.04, 0.02 | 0.442 |
| 2T | Total effects of Land use mix diversity^1^ | **-5.06** | **-10.09, -0.04** | **0.048** |
| 2D | Direct effects of Land use mix diversity^1^ | **-7.32** | **-13.48, -1.17** | **0.020** |
| 3T | Total effects of Transit stop proximity | 1.85 | -1.92, 5.62 | 0.335 |
| 3D | Direct effects of Transit stop proximity | 3.70 | -0.47, 7.88 | 0.082 |
| 4T | Total effects of Recreation facilities^2^ | -3.25 | -8.06, 1.56 | 0.185 |
| 4D | Direct effects of Recreation facilities^2^ | -0.53 | -6.23, 5.16 | 0.854 |
| 5T | Total effects of Park proximity | -2.81 | -6.49, 0.87 | 0.135 |
| 5D | Direct effects of Park proximity | -0.42 | -4.86, 4.03 | 0.854 |
| 6T | Total effects of Accessibility and walking facilities | 0.03 | -7.21, 7.28 | 0.993 |
| 6D | Direct effects of Accessibility and walking facilities | 2.85 | -5.10, 10.79 | 0.483 |
| 7T | Total effects of Traffic safety | **-13.97** | **-20.13, -7.81** | **<0.001** |
| 7D | Direct effects of Traffic safety | **-11.98** | **-18.46, -5.50** | **<0.001** |
| 8T | Total effects of Pedestrians infrastructure and safety | 0.44 | -5.51, 6.38 | 0.885 |
| 8D | Direct effects of Pedestrians infrastructure and safety | 5.19 | -1.33, 11.71 | 0.119 |
| 9T | Total effects of Crime safety | **-5.25** | **-9.95, -0.55** | **0.028** |
| 9D | Direct effects of Crime safety | -2.18 | -7.09, 2.72 | 0.383 |
| 10T | Total effects of Aesthetics | -5.16 | -10.51, 0.18 | 0.058 |
| 10D | Direct effects of Aesthetics | -4.75 | -10.36, 0.86 | 0.097 |
| 11T | Total effects of personal electronic devices | **27.06** | **22.15, 31.97** | **<0.001** |
| 11D | Direct effects of personal electronic devices | **27.06** | **22.15, 31.97** | **<0.001** |
| 12T | Total effects of having own social media | **39.58** | **29.19, 49.97** | **<0.001** |
| 12D | Direct effects of having own social media | **39.58** | **29.19, 49.97** | **<0.001** |
| 13T | Total effects of electronic devices in the bedroom | **16.56** | **14.03, 19.10** | **<0.001** |
| 13D | Direct effects of electronic thing in the bedroom | **16.56** | **14.03, 19.10** | **<0.001** |

*Note.* ^1^ excluding transit stops; ^2^ excluding parks; *b* = regression coefficient; CI = confidence intervals; in bold: effects significant at *p*<0.05

**Table S3. Adolescents’ sex as a moderator of total and direct effects of perceived neighbourhood and home environment characteristics on adolescents’ screen time**

| Model | Environmental effect estimated | Regression coefficient | ***b*** | **95% CI** | ***p*** |
| --- | --- | --- | --- | --- | --- |
| 1IT* | Total effects of Residential density | Interaction with sex | **0.08** | **0.05, 0.12** | **<0.001** |
|  |  | Males | **-0.06** | **-0.09, -0.02** | **0.001** |
|  |  | Females | 0.02 | -0.01, 0.06 | 0.117 |
| 1ID* | Direct effects of Residential density | Interaction with sex | **0.08** | **0.04,0.11** | **<0.001** |
|  |  | Males | **-0.06** | **-0.09, -0.02** | **0.001** |
|  |  | Females | 0.02 | -0.01, 0.05 | 0.182 |
| 2IT* | Total effects of Land use mix diversity^1^ | Interaction with sex | 5.89 | -2.81, 14.59 | 0.185 |
| 2ID* | Direct effects of Land use mix diversity^1^ | Interaction with sex | 8.27 | -0.37, 16.90 | 0.061 |
| 3IT* | Total effects of Transit stop proximity | Interaction with sex | 1.20 | -5.51, 7.91 | 0.725 |
| 3ID* | Direct effects of Transit stop proximity | Interaction with sex | 1.53 | -5.17, 8.23 | 0.654 |
| 4IT* | Total effects of Recreation facilities^2^ | Interaction with sex | -0.12 | -8.76, 8.52 | 0.978 |
| 4ID* | Direct effects of Recreation facilities^2^ | Interaction with sex | 1.19 | -7.50, 9.89 | 0.788 |
| 5IT* | Total effects of Park proximity | Interaction with sex | 5.38 | -0.78, 11.54 | 0.087 |
| 5ID* | Direct effects of Park proximity | Interaction with sex | **6.49** | **0.30, 12.68** | **0.040** |
|  |  | Males | -3.99 | -9.57, 1.59 | 0.161 |
|  |  | Females | 2.50 | -2.72, 7.73 | 0.348 |
| 6IT* | Total effects of Accessibility and walking facilities | Interaction with sex | -1.70 | -14.84, 11.44 | 0.800 |
| 6ID* | Direct effects of Accessibility and walking facilities | Interaction with sex | 1.85 | -11.36, 15.06 | 0.784 |
| 7IT* | Total effects of Traffic safety | Interaction with sex | -4.39 | -15.81, 7.03 | 0.451 |
| 7ID* | Direct effects of Traffic safety | Interaction with sex | -4.08 | -15.52, 7.37 | 0.485 |
| 8IT* | Total effects of Pedestrians infrastructure | Interaction with sex | 0.85 | -10.69, 12.40 | 0.885 |
| 8ID* | Direct effects of Pedestrians infrastructure | Interaction with sex | 3.35 | -8.32, 15.02 | 0.573 |
| 9IT* | Total effects of Crime safety | Interaction with sex | 5.24 | -3.03, 13.52 | 0.214 |
| 9ID* | Direct effects of Crime safety | Interaction with sex | 4.32 | -4.01, 12.64 | 0.310 |
| 10IT* | Total effects of Aesthetics | Interaction with sex | -3.93 | -13.18, 5.33 | 0.406 |
| 10ID* | Direct effects of Aesthetics | Interaction with sex | -4.96 | -14.41,4.48 | 0.303 |
| 11IT* | Total effects of personal electronic devices | Interaction with sex | -0.38 | -8.74, 7.98 | 0.928 |
| 11ID* | Direct effects of personal electronic devices | Interaction with sex | -0.38 | -8.74, 7.98 | 0.928 |
| 12IT* | Total effects of having own social media | Interaction with sex | -10.83 | -29.48, 7.82 | 0.255 |
| 12ID* | Direct effects of having own social media | Interaction with sex | -10.83 | -29.48, 7.82 | 0.255 |
| 13IT* | Total effects of electronic devices in the bedroom | Interaction with sex | -1.98 | -6.66, 2.70 | 0.407 |
| 13ID* | Direct effects of electronic devices in the bedroom | Interaction with sex | -1.98 | -6.66, 2.70 | 0.407 |

*Note.* ^1^ excluding transit stops; ^2^ excluding parks; GAMM, generalised additive mixed model. *b* = regression coefficient; CI = confidence interval; in bold: effects significant at *p*<0.05

**Table S4. City as a moderator of total effects of perceived home environment characteristics on adolescents’ screen time**

|  | Electronic devices in the bedroom | | | Having own social media | | |
| --- | --- | --- | --- | --- | --- | --- |
| City (country) | ***b*** | **95% CI** | ***p*** | ***b*** | **95% CI** | ***p*** |
| *High-income countries* |  |  |  |  |  |  |
| Melbourne (AUS) | 12.90 | -0.04, 25.84 | 0.051 | **99.30** | **36.49, 162.12** | **0.002** |
| Ghent (BEL) | **26.15** | **14.58, 37.73** | **<0.001** | 8.60 | -31.02, 48.22 | 0.670 |
| Hradec Králové (CZE) | 11.56 | -7.30, 30.42 | 0.230 | 8.94 | -81.00, 98.87 | 0.846 |
| Olomouc (CZE) | **24.36** | **2.58, 46.13** | **0.028** | **87.74** | **10.00, 165.49** | **0.027** |
| Odense (DNK) | **24.64** | **10.86, 38.43** | **<0.001** | 28.45 | -18.80, 75.70 | 0.238 |
| Hong Kong (HKG, China) | **11.35** | **6.29, 16.42** | **<0.001** | 3.06 | -25.22, 31.35 | 0.832 |
| Haifa (ISR) | 8.28 | -3.59, 20.15 | 0.172 | 51.25 | -3.00, 105.50 | 0.064 |
| Various cities (PRT) | -1.08 | -16.85, 14.68 | 0.893 | 32.17 | -62.00, 126.34 | 0.503 |
| Valencia (ESP) | 5.94 | -2.60, 14.49 | 0.173 | 22.62 | -10.74, 55.99 | 0.184 |
| Baltimore (USA) | **24.82** | **17.04, 32.60** | **<0.001** | **48.42** | **18.77, 78.06** | **<0.001** |
| Seattle (USA) | **19.05** | **10.48, 27.63** | **<0.001** | -0.35 | -29.50, 28.80 | 0.981 |
| *Low-middle-income countries* |  |  |  |  |  |  |
| Dhaka (BGD) | **37.36** | **19.71, 55.01** | **<0.001** | **104.20** | **40.52, 167.89** | **<0.001** |
| Curitiba (BRA) | **15.20** | **7.39, 23.01** | **<0.001** | **100.99** | **53.28, 148.70** | **<0.001** |
| Chennai (IND) | 9.85 | -3.67, 23.37 | 0.153 | **49.32** | **14.74, 83.90** | **0.005** |
| Kuala Lumpur (MYS) | **16.18** | **5.62, 26.73** | **0.003** | 18.81 | -17.11, 54.73 | 0.305 |
| Gombe (NGA) | **38.74** | **28.34, 49.13** | **<0.001** | **120.76** | **85.65, 155.86** | **<0.001** |

*Note.* *b* = regression coefficient; CI = confidence interval; in bold: effects significant at *p*<0.05

**Summary of findings:**

Parent-perceived land use mix – diversity and traffic safety were negatively associated with adolescents’ screen time (Table S2). For these neighbourhood attributes, both total and direct effects were statistically significant. The total effect of perceived crime safety on screen time was also negative. All home environmental attributes examined in this study, including having own social media, personal electronic devices and electronic devices in the bedroom, were positively related to screen time in pooled analyses.

Adolescent sex moderated the associations of residential density and park proximity with screen time (Table S3). Specifically, only males showed a negative association between parent-perceived residential density and screen time. They also tended to show a negative association between park proximity and screen time (compared to females). Having own social media and electronic devices in the bedroom were the only environmental attributes with city-specific effects on screen time (Table S4).

Significant positive associations between the former attribute and screen time were observed in Baltimore (USA), Gombe (Nigeria), Olomouc (Czech Republic), Curitiba (Brazil), Melbourne (Australia), Dhaka (Bangladesh) and Chennai (India); and a weaker positive association was found in Haifa (Israel). There was no sufficient evidence supporting an association between these variables in the other cities. As to electronic devices in the bedroom, the city-specific associations were more consistent. Significant positive associations were found in 10 of the 16 cities. Weaker evidence for a positive association was also observed in Melbourne (Australia). Insufficient support of an association between electronic devices in the bedroom and screen time was observed in Valencia (Spain), Portugal, Hradec Králové (Czech Republic), Chennai (India) and Haifa (Israel).


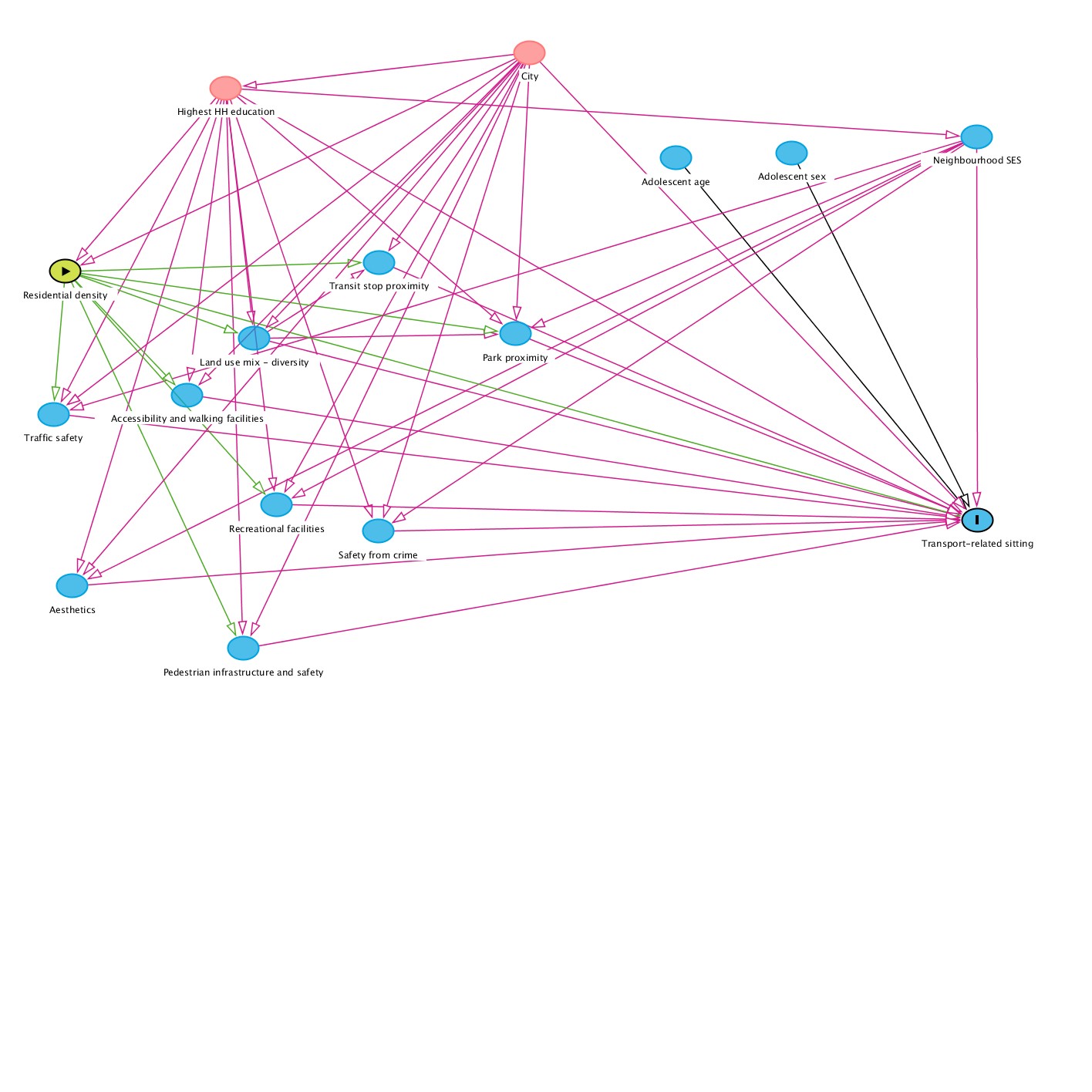


**Figure S2.** Directed acyclic graph (DAG) depicting the hypothesised relations between characteristics of the neighbourhood environment with transport-related sitting time. Through the DAG, we identified which covariates to include in the statistical analyses to sufficiently control for potential confounders. This particular DAG was used to inform the model of the total effect of parental perceptions of neighbourhood residential density on adolescents’ transport-related sitting time. Variables with red circles denote the set of potential confounders. A minimal sufficient set of confounders (included in the regression models) is a subset of this set of variables.

**Table S5. Covariates of regression models of perceived neighbourhood environment characteristics (exposures) and adolescents’ transport-related sitting time (outcome)**

| **Model** | **Effect estimated** | **Covariates** |
| --- | --- | --- |
| 1T | Total effect of Residential density | adolescent age; adolescent sex; area-level SES; highest education in household; city |
| 1D | Direct effect of Residential density | *accessibility and walking facilities*; adolescent age; adolescent sex; city; highest education in household; *land use mix – diversity*; area-level SES; *pedestrian infrastructure and safety*; *recreation facilities*; *park proximity*; *traffic safety*; *transit stop proximity* |
| 2T | Total effect of Land use mix diversity^1^ | adolescent age; adolescent sex; area-level SES; highest education in household; city; residential density |
| 2D | Direct effect of Land use mix diversity^1^ | residential density; parks; transit stops; adolescent age; adolescent sex; highest education in household; city; area-level SES |
| 3T | Total effect of Transit stop proximity | residential density; land use mix - diversity; adolescent age; adolescent sex; city; highest education in household; area-level SES |
| 3D | Direct effect of Transit stop proximity | residential density; land use mix - diversity; adolescent age; adolescent sex; city; highest education in household; area-level SES |
| 4T | Total effect of Recreation facilities^2^ | adolescent age; adolescent sex; area-level SES; highest education in household; city; residential density |
| 4D | Direct effect of Recreation facilities^2^ | adolescent age; adolescent sex; area-level SES; highest education in household; city; residential density |
| 5T | Total effect of Park proximity | residential density; land use mix - diversity; adolescent age; adolescent sex; city; highest education in household; area-level SES |
| 5D | Direct effect of Park proximity | residential density; land use mix - diversity; adolescent age; adolescent sex; city; highest education in household; area-level SES |
| 6T | Total effect of Accessibility and walking facilities | adolescent age; adolescent sex; area-level SES; highest education in household; city; residential density |
| 6D | Direct effect of Accessibility and walking facilities | adolescent age; adolescent sex; area-level SES; highest education in household; city; residential density |
| 7T | Total effect of Traffic safety | adolescent age; adolescent sex; area-level SES; highest education in household; city; residential density |
| 7D | Direct effect of Traffic safety | adolescent age; adolescent sex; area-level SES; highest education in household; city; residential density |
| 8T | Total effect of Pedestrian infrastructure and safety | adolescent age; adolescent sex; area-level SES; highest education in household; city; residential density |
| 8D | Direct effect of Pedestrian infrastructure and safety | adolescent age; adolescent sex; area-level SES; highest education in household; city; residential density |
| 9T | Total effect of Crime safety | adolescent age; adolescent sex; area-level SES highest education in household; city |
| 9D | Direct effect of Crime safety | adolescent age; adolescent sex; area-level SES; highest education in household; city |
| 10T | Total effect of Aesthetics | adolescent age; adolescent sex; area-level SES; highest education in household; city |
| 10D | Direct effect of Aesthetics | adolescent age; adolescent sex; area-level SES; highest education in household; city |

*Note.* ^1^ excluding transit stops; ^2^ excluding parks; SES, socio-economic status. Potential mediators in *italics*.

**Table S6. Total and direct effects of perceived neighbourhood environment characteristics on adolescents’ transport-related sitting time [complete case analyses; N=4975]**

| **Model** | **Effect estimated** | ***e^b^*** | **95% CI** | ***p*-value** |
| --- | --- | --- | --- | --- |
| 1T | Total effect on Residential density | 0.9998 | 0.9995, 1.0000 | 0.076 |
| 1D | Direct effect of Residential density | 0.9999 | 0.9996, 1.001 | 0.306 |
| 2T | Total effect of Land use mix diversity^1^ | **0.90** | **0.86, 0.94** | **<0.001** |
| 2D | Direct effect of Land use mix diversity^1^ | **0.90** | **0.85, 0.95** | **<0.001** |
| 3T | Total effect of Transit stop proximity | 0.98 | 0.94, 1.02 | 0.293 |
| 3D | Direct effect of Transit stop proximity | 0.98 | 0.94, 1.02 | 0.293 |
| 4T | Total effect of Recreation facilities^2^ | **0.95** | **0.91, 1.00** | **0.035** |
| 4D | Direct effect of Recreation facilities^2^ | **0.95** | **0.91, 1.00** | **0.035** |
| 5T | Total effect of Park proximity | 1.01 | 0.97, 1.05 | 0.592 |
| 5D | Direct effect of Park proximity | 1.01 | 0.97, 1.05 | 0.592 |
| 6T | Total effect of Accessibility and walking facilities | **0.89** | **0.84, 0.94** | **<0.001** |
| 6D | Direct effect of Accessibility and walking facilities | **0.89** | **0.84, 0.94** | **<0.001** |
| 7T | Total effect of Traffic safety | 0.98 | 0.92, 1.03 | 0.396 |
| 7D | Direct effect of Traffic safety | 0.98 | 0.92, 1.03 | 0.396 |
| 8T | Total effect of Pedestrian infrastructure and safety | **0.94** | **0.89, 0.99** | **0.017** |
| 8D | Direct effect of Pedestrian infrastructure and safety | **0.94** | **0.89, 0.99** | **0.017** |
| 9T | Total effect of Crime safety | 0.96 | 0.92, 1.00 | 0.061 |
| 9D | Direct effect of Crime safety | 0.96 | 0.92, 1.00 | 0.061 |
| 10T | Total effect of Aesthetics | 1.002 | 0.961, 1.051 | 0.926 |
| 10D | Direct effect of Aesthetics | 1.002 | 0.961, 1.051 | 0.926 |

*Note.* ^1^ excluding transit stops; ^2^ excluding parks; e^b^ = exponentiated regression coefficient; CI = confidence intervals; in bold: effects significant at *p*<0.05

**Table S7. Adolescents’ sex as a moderator of total and direct effects of perceived neighbourhood environment characteristics on adolescents’ transport-related sitting time**

| **Model** | **Effect estimated** | **Moderator** | ***e^b^*** | **95% CI** | ***p*-value** |
| --- | --- | --- | --- | --- | --- |
| 1T | Total effects of Residential density | Interaction with sex  Males  Females | **0.9997**  0.99997  **0.9996** | **0.999, 1.00004**  0.9997, 1.0003  **0.9994, 0.9999** | **0.028**  0.858  **0.008** |
| 1D | Direct effects of Residential density | Interaction with sex  Males  Females | **0.9997**  0.99995  0.9997 | **0.9990, 0.9999**  0.9997,1.0004  0.9994, 1.00001 | **0.028**  0.773  0.063 |
| 2T | Total effects of Land use mix diversity^1^ | Interaction with sex | 0.95 | 0.88, 1.02 | 0.163 |
| 2D | Direct effects of Land use mix diversity^1^ | Interaction with sex | 0.95 | 0.88, 1.03 | 0.218 |
| 3T | Total effects of Transit stop proximity | Interaction with sex  Males  Females | **1.06**  **0.95**  1.01 | **1.00, 1.12**  **0.90, 0.96**  0.96, 1.05 | **0.050**  **0.033**  0.762 |
| 3D | Direct effects of Transit stop proximity | Interaction with sex Males  Females | **1.06**  **0.95**  1.01 | **1.00, 1.13**  **0.90, 0.97**  0.96, 1.05 | **0.050**  **0.033**  0.762 |
| 4T | Total effects of Recreation facilities^2^ | Interaction with sex | 0.98 | 0.91, 1.15 | 0.568 |
| 4D | Direct effects of Recreation facilities^2^ | Interaction with sex | 0.98 | 0.91, 1.15 | 0.568 |
| 5T | Total effects of Park proximity | Interaction with sex | 1.02 | 0.96, 1.07 | 0.484 |
| 5D | Direct effects of Park proximity | Interaction with sex | 1.02 | 0.96, 1.07 | 0.484 |
| 6T | Total effects of Accessibility and walking facilities | Interaction with sex | 0.98 | 0.87, 1.09 | 0.726 |
| 6D | Direct effects of Accessibility and walking facilities | Interaction with sex | 0.98 | 0.87, 1.09 | 0.726 |
| 7T | Total effects of Traffic safety | Interaction with sex | 1.02 | 0.92, 1.13 | 0.724 |
| 7D | Direct effects of Traffic safety | Interaction with sex | 1.02 | 0.92, 1.13 | 0.724 |
| 8T | Total effect of Pedestrian infrastructure and safety | Interaction with sex | 0.92 | 0.84, 1.02 | 0.128 |
| 8D | Direct effect of Pedestrian infrastructure and safety | Interaction with sex | 0.92 | 0.84, 1.02 | 0.128 |
| 9T | Total effects of Accessibility and walking facilities | Interaction with sex | 0.98 | 0.87, 1.09 | 0.726 |
| 9D | Direct effects of Accessibility and walking facilities | Interaction with sex | 0.98 | 0.87, 1.09 | 0.726 |
| 10T | Total effects of Aesthetics | Interaction with sex | 1.01 | 0.93, 1.09 | 0.858 |
| 10D | Direct effects of Aesthetics | Interaction with sex | 1.01 | 0.93, 1.09 | 0.858 |

*Note.* ^1^ excluding transit stops; ^2^ excluding parks; e^b^ = exponentiated regression coefficient; CI = confidence intervals; in bold: effects significant at *p*<0.05

**Table S8.** **City as a moderator of the total and direct effects of neighbourhood environment characteristics on adolescents’ transport-related sitting**

|  | **Land use mix diversity^1^** | | | **Accessibility and walking facilities** | | | | T**raffic safety** | | | |
| --- | --- | --- | --- | --- | --- | --- | --- | --- | --- | --- | --- |
| **City (country)** | ***e^b^*** | **95% CI** | *p* | ***e^b^*** | **95% CI** | *p* | | ***e^b^*** | **95% CI** | | *p* |
| *High-income countries* |  |  |  |  |  |  | |  |  |  | |
| Melbourne (AUS) | 0.86 | 0.71, 1.05 | 0.148 | 0.84 | 0.59, 1.17 | 0.294 | | 0.85 | 0.64, 1.12 | 0.253 | |
| Ghent (BEL) | **0.81** | **0.96, 0.98** | **0.032** | 0.84 | 0.64, 1.09 | 0.189 | | 0.76 | 0.58, 1.00 | 0.052 | |
| Hradec Králové (CZE) | 0.95 | 0.71, 1.27 | 0.723 | 0.63 | 0.36, 1.09 | 0.103 | | 1.21 | 0.78, 1.90 | 0.396 | |
| Olomouc (CZE) | 1.13 | 0.81, 1.55 | 0.480 | 0.79 | 0.48, 1.31 | 0.369 | | 1.36 | 0.86, 2.18 | 0.188 | |
| Odense (DNK) | **0.81** | **0.66, 0.98** | **0.028** | **0.65** | **0.42, 0.95** | **0.028** | | 1.21 | 0.94, 1.55 | 0.144 | |
| Hong Kong (HKG, CHN) | **0.88** | **0.80, 0.96** | **0.003** | **0.75** | **0.65, 0.85** | **<0.001** | | 0.88 | 0.77, 1.00 | 0.056 | |
| Haifa (ISR) | 0.89 | 0.73, 1.08 | 0.235 | 1.05 | 0.76, 1.45 | 0.781 | | 1.07 | 0.85, 1.35 | 0.555 | |
| Various cities (PRT) | 0.79 | 0.59, 1.05 | 0.106 | 0.57 | 0.32, 1.02 | 0.060 | | 1.02 | 0.63, 1.65 | 0.944 | |
| Valencia (ESP) | **0.70** | **0.57, 0.89** | **0.002** | 0.91 | 0.70, 1.20 | 0.505 | | 0.97 | 0.83, 1.13 | 0.684 | |
| Baltimore (USA) | 0.97 | 0.85, 1.12 | 0.704 | 0.95 | 0.77, 1.19 | 0.676 | | 1.13 | 0.91, 1.40 | 0.272 | |
| Seattle (USA) | 0.96 | 0.84, 1.10 | 0.585 | 0.92 | 0.77, 1.12 | 0.423 | | 0.98 | 0.79, 1.22 | 0.877 | |
| *Low-middle-income countries* |  |  |  |  |  |  | |  |  |  | |
| Dhaka (BGD) | **0.66** | **0.44, 0.98** | **0.041** | 1.11 | 0.72, 1.70 | 0.654 | | 1.26 | 0.83, 1.92 | 0.286 | |
| Curitiba (BRA) | 0.97 | 0.82, 1.04 | 0.715 | 0.91 | 0.76, 1.08 | 0.299 | | 1.02 | 0.88, 1.17 | 0.788 | |
| Chennai (IND) | **0.71** | **0.58, 0.87** | **0.001** | 1.00 | 0.80, 1.26 | 0.989 | | 1.01 | 0.83, 1.25 | 0.908 | |
| Kuala Lumpur (MYS) | 1.00 | 0.84, 1.19 | 0.957 | 1.15 | 0.85, 1.57 | 0.361 | | 1.04 | 0.81, 1.32 | 0.771 | |
| Gombe (NGA) | 1.08 | 0.89, 1.34 | 0.408 | 1.15 | 0.90, 1.45 | 0.260 | | 0.84 | 0.70, 1.01 | 0.057 | |
|  | | | | | | | | | | | |
|  | **Pedestrian infrastructure** | | | **Aesthetics** | | | | **Transit stop proximity** | | | |
| **Country - city** | ***e^b^*** | **95% CI** | *p* | ***e^b^*** | **95% CI** | | *p* | ***e^b^*** | **95% CI** | | *p* |
| *High-income countries* |  |  |  |  |  | |  |  |  | |  |
| Melbourne (AUS) | 0.89 | 0.64, 1.22 | 0.452 | 0.87 | 0.70,1.08 | | 0.210 | 0.82 | 0.66, 1.02 | | 0.076 |
| Ghent (BEL) | 0.87 | 0.66, 1.15 | 0.335 | **0.73** | **0.58, 0.93** | | **0.010** | 1.00 | 0.80, 1.25 | | 0.977 |
| Hradec Králové (CZE) | 0.84 | 0.55, 1.27 | 0.412 | 1.19 | 0.78, 1.80 | | 0.434 | 1.05 | 0.84, 1.31 | | 0.631 |
| Olomouc (CZE) | 0.97 | 0.57, 1.65 | 0.916 | 1.07 | 0.74, 1.63 | | 0.645 | 1.32 | 0.99, 1.77 | | 0.058 |
| Odense (DNK) | **0.72** | **0.56, 0.92** | **0.009** | 0.81 | 0.64, 1.03 | | 0.084 | 1.13 | 0.88, 1.46 | | 0.351 |
| Hong Kong (HKG, CHN) | **0.82** | **0.73, 0.92** | **0.001** | 1.00 | 0.90, 1.11 | | 0.968 | 0.96 | 0.90, 1.03 | | 0.260 |
| Haifa (ISR) | 0.95 | 0.76, 1.19 | 0.636 | 1.01 | 0.83, 1.23 | | 0.919 | 0.99 | 0.82, 1.20 | | 0.902 |
| Various cities (PRT) | 0.84 | 0.53, 1.30 | 0.424 | 0.90 | 0.59, 1.36 | | 0.632 | 1.13 | 0.85, 1.52 | | 0.394 |
| Valencia (ESP) | **0.80** | **0.67, 0.97** | **0.021** | 0.88 | 0.75, 1.02 | | 0.088 | 1.04 | 0.84, 1.27 | | 0.720 |
| Baltimore (USA) | 0.97 | 0.80, 1.17 | 0.750 | 1.03 | 0.89, 1.21 | | 0.659 | 0.99 | 0.90, 1.08 | | 0.841 |
| Seattle (USA) | 1.00 | 0.83, 1.02 | 0.969 | 1.09 | 0.91, 1.30 | | 0.340 | 0.98 | 0.89, 1.09 | | 0.734 |
| *Low-middle-income countries* |  |  |  |  |  | |  |  |  | |  |
| Dhaka (BGD) | 0.74 | 0.48, 1.34 | 0.175 | 1.01 | 0.73, 1.40 | | 0.937 | **2.14** | **1.73, 2.16** | | **<0.001** |
| Curitiba (BRA) | 0.89 | 0.77, 1.02 | 0.452 | 1.004 | 0.89, 1.14 | | 0.955 | 1.20 | 0.997, 1.43 | | 0.053 |
| Chennai (IND) | **1.34** | **1.12, 1.58** | **0.001** | **1.22** | **1.03, 1.45** | | **0.019** | **0.88** | **0.77, 0.998** | | **0.047** |
| Kuala Lumpur (MYS) | 1.09 | 0.88, 1.38 | 0.411 | 1.01 | 0.83, 1.22 | | 0.943 | 0.93 | 0.98, 1.03 | | 0.147 |
| Gombe (NGA) | **1.22** | **1.003, 1.48** | **0.047** | **1.21** | **1.01, 1.45** | | **0.045** | 1.07 | 0.91, 1.26 | | 0.384 |

*Note.* ^1^ excluding transit stops; e^b^ = exponentiated regression coefficient; CI = confidence interval; AUS, Australia; BEL, Belgium; CZE, Czech Republic; DNK, Denmark; HKG, Hong Kong; CHN, China; ISR, Israel; PRT, Portugal; ESP, Spain; USA, Unites States of America; BGD, Bangladesh; BRA, Brazil; IND, India; MYS, Malaysia; NGA, Nigeria The estimates above are estimates of total and direct effects for all environmental characteristics except for land use mix – diversity for which they represent total effects only. Statistically significant effects (*p*<.05) are in bold.

**Summary of findings:**

Parent-perceived land use mix – diversity, access to recreation facilities, accessibility and walking facilities, and pedestrian infrastructure and safety were all negatively associated with adolescents’ transport-related sitting time (Table S6). For these neighbourhood attributes, both total and direct effects were statistically significant.

Adolescent sex moderated the associations of residential density and transit stop proximity with transport-related sitting time (Table S7). Specifically, only females showed a negative association between residential density and transport-related sitting time in the total-effect models. In contrast, only males showed negative associations between transit stop proximity and transport-related sitting time.

Between-city differences in parent-perceived environmental correlates of adolescents’ transport-related sedentary time were observed for six environmental attributes: land use mix – diversity, accessibility and walking facilities, traffic safety, pedestrian infrastructure, aesthetics and transit stop proximity (Table S8). Negative associations between land use mix – proximity and transport-related sitting were observed in Ghent (Belgium), Valencia (Spain), Odense (Denmark), Hong Kong (China), Dhaka (Bangladesh) and Chennai (India). Accessibility and walking facilities were negatively related to the same outcome in Odense (Denmark) and Hong Kong (China), with Portuguese adolescents also tending to show a negative association (Table S8). Although not statistically significant, the associations between traffic safety and transport-related sitting time in Gombe (Nigeria), Ghent (Belgium), and Hong Kong (China) tended to be negative. Pedestrian infrastructure was negatively related to transport-related sitting time in Valencia (Spain), Odense (Denmark) and Hong Kong (China), and positively related to the same outcome in Gombe (Nigeria) and Chennai (India). Similarly, aesthetics was positively associated with transport-related sitting time in Gombe (Nigeria) and Chennai (India). However, it showed a negative association in adolescents from Ghent (Belgium) and marginally negative associations in Valencia (Spain) and Odense (Denmark). Having closer transit stops from home was related to less transport-related sitting time in Chennai (India) and, marginally, in Melbourne (Australia), while the opposite was true for Dhaka (Bangladesh) and, marginally, Olomouc (Czech Republic) and Curitiba (Brazil) (Table S8).


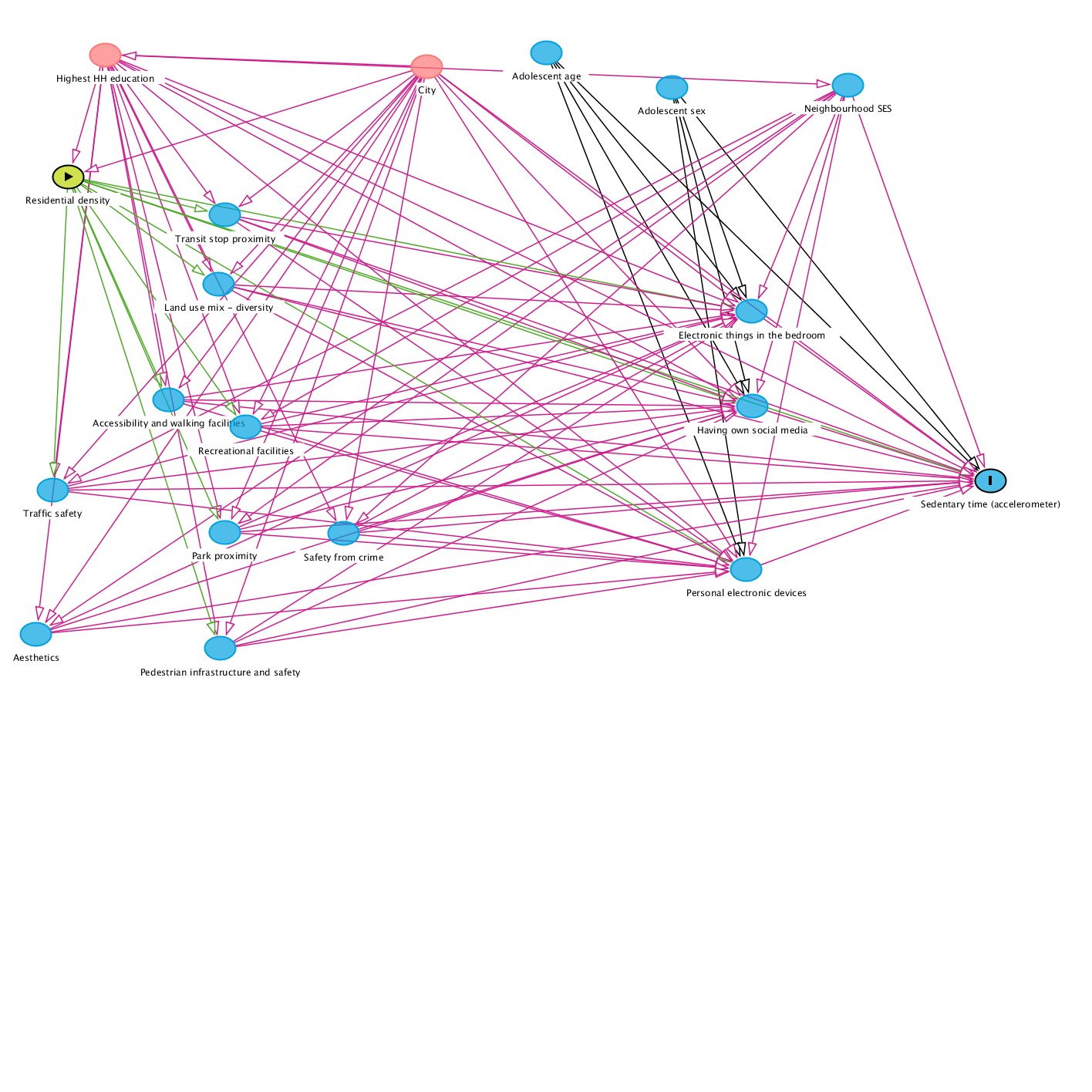


**Figure S3.** Directed acyclic graph (DAG) depicting the hypothesised relations between characteristics of the neighbourhood and home environments with accelerometer-assessed sedentary time. Through the DAG, we identified which covariates to include in the statistical analyses to sufficiently control for potential confounders. This particular DAG was used to inform the model of the total effect of parental perceptions of neighbourhood residential density on adolescents’ accelerometer-assessed sedentary time. Variables with red circles denote the set of potential confounders. A minimal sufficient set of confounders (included in the regression models) is a subset of this set of variables.

**Table S9. Covariates of regression models of perceived neighbourhood and home environment characteristics (exposures) and adolescents’ accelerometer-assessed sedentary time (outcome)**

| **Model** | **Effect estimated** | **Covariates** |
| --- | --- | --- |
| 1T | Total effects on Residential density | adolescent age; adolescent sex; area-level SES; highest education in household; city; valid days of wear; average wear time |
| 1D | Direct effects of Residential density | *accessibility and walking facilities*; adolescent age; adolescent sex; aesthetics; city; *electronic devices in the bedroom*; *having own social media*; highest education in household; *land use mix – diversity^1^*; area-level SES; *park proximity*; *pedestrian infrastructure and safety*; *personal electronic devices*; *recreation facilities^2^*; crime safety; *traffic safety*; *transit stop proximity*; valid days of wear; average wear time |
| 2T | Total effects of Land use mix diversity^1^ | adolescent age; adolescent sex; area-level SES; highest education in household; city; residential density; valid days of wear; average wear time |
| 2D | Direct effects of Land use mix diversity^1^ | accessibility and walking facilities; adolescent age; adolescent sex; aesthetics; city; *electronic devices in the bedroom*; *having own social media*; highest education in household; area-level SES; park proximity; pedestrian infrastructure and safety; *personal electronic devices*; recreation facilities^2^; residential density; crime safety; traffic safety; transit stop proximity; valid days of wear; average wear time |
| 3T | Total effects of Transit stop proximity | adolescent age; adolescent sex; area-level SES; highest education in household; city; residential density; valid days of wear; average wear time |
| 3D | Direct effects of Transit stop proximity | accessibility and walking facilities; adolescent age; adolescent sex; aesthetics; city; *electronic devices in the bedroom*; *having own social media*; highest education in household; residential density; area-level SES; pedestrian infrastructure and safety; *personal electronic devices*; recreation facilities^2^; crime safety; traffic safety; valid days of wear; average wear time |
| 4T | Total effects of Recreation facilities^2^ | adolescent age; adolescent sex; area-level SES; highest education in household; city; residential density; valid days of wear; average wear time |
| 4D | Direct effects of Recreation facilities^2^ | accessibility and walking facilities; adolescent age; adolescent sex; aesthetics; city; *electronic devices in the bedroom*; *having own social media*; highest education in household; land use mix - diversity^1^; area-level SES; park proximity; pedestrian infrastructure and safety; *personal electronic devices*; residential density; crime safety; traffic safety; transit stop proximity |
| 5T | Total effects of Park proximity | adolescent age; adolescent sex; area-level SES; highest education in household; city; residential density; valid days of wear; average wear time |
| 5D | Direct effects of Park proximity | accessibility and walking facilities; adolescent age; adolescent sex; aesthetics; city; *electronic devices in the bedroom*; *having own social media*; highest education in household; land use mix - diversity^1^; area-level SES; pedestrian infrastructure and safety; *personal electronic devices*; residential density; crime safety; traffic safety; valid days of wear; average wear time |
| 6T | Total effects of Accessibility and walking facilities | adolescent age; adolescent sex; area-level SES; highest education in household; city; residential density; valid days of wear; average wear time |
| 6D | Direct effects of Accessibility and walking facilities | adolescent age; adolescent sex; aesthetics; city; *electronic devices in the bedroom*; *having own social media*; highest education in household; land use mix - diversity^1^; area-level SES; park proximity; pedestrian infrastructure and safety; *personal electronic devices*; recreation facilities^2^; residential density; crime safety; traffic safety; transit stop proximity; valid days of wear; average wear time |
| 7T | Total effects of Traffic safety | adolescent age; adolescent sex; area-level SES; highest education in household; city; residential density; valid days of wear; average wear time |
| 7D | Direct effects of Traffic safety | accessibility and walking facilities; adolescent age; adolescent sex; aesthetics; city; *electronic devices in the bedroom*; *having own social media*; highest education in household; land use mix - diversity^1^; area-level SES; park proximity; pedestrian infrastructure and safety; *personal electronic devices*; recreation facilities^2^; residential density; crime safety; transit stop proximity; valid days of wear; average wear time |
| 8T | Total effects of Pedestrians infrastructure and safety | adolescent age; adolescent sex; area-level SES; highest education in household; city; residential density; valid days of wear; average wear time |
| 8D | Direct effects of Pedestrians infrastructure and safety | accessibility and walking facilities; adolescent age; adolescent sex; aesthetics; city; *electronic devices in the bedroom*; *having own social media*; highest education in household; land use mix - diversity^1^; area-level SES; park proximity; *personal electronic devices*; recreation facilities^2^; residential density; crime safety; traffic safety; transit stop proximity; valid days of wear; average wear time |
| 9T | Total effects of Crime safety | adolescent age, adolescent sex, area-level SES; Highest education in household, city; valid days of wear; average wear time |
| 9D | Direct effects of Crime safety | accessibility and walking facilities; adolescent age; adolescent sex; city; *electronic devices in the bedroom*; *having own social media*; highest education in household; land use mix - diversity^1^; area-level SES, park proximity; pedestrian infrastructure and safety; *personal electronic devices*; recreation facilities^2^; residential density; crime safety; traffic safety; transport stop proximity; valid days of wear; average wear time |
| 10T | Total effects of Aesthetics | adolescent age; adolescent sex; area-level SES; highest education in household; city; valid days of wear; average wear time |
| 10D | Direct effects of Aesthetics | accessibility and walking facilities; adolescent age; adolescent sex; city; *electronic devices in the bedroom*; *having own social media*; highest education in household; land use mix - diversity^1^; area-level SES; pedestrian infrastructure and safety; *personal electronic devices*; recreation facilities^2^; residential density; crime safety; traffic safety; transit stop proximity; valid days of wear; average wear time |
| 11T | Total effects of Personal electronic devices | accessibility and walking facilities; adolescent age; adolescent sex; aesthetics; city; highest education in household; land use mix - diversity^1^; area-level SES; park proximity; pedestrian infrastructure and safety; residential density; recreation facilities^2^; crime safety; traffic safety; transit stop proximity; valid days of wear; average wear time |
| 11D | Direct effects of Personal electronic devices | accessibility and walking facilities; adolescent age; adolescent sex; aesthetics; city; electronic devices in the bedroom; having own social media; highest education in household; land use mix – diversity^1^; area-level SES; pedestrian infrastructure and safety; residential density; recreation facilities^2^; crime safety; traffic safety; valid days of wear; average wear time |
| 12T | Total effects of Having own social media | accessibility and walking facilities; adolescent age; adolescent sex; aesthetics; city; highest education in household; land use mix - diversity^1^; area-level SES; pedestrian infrastructure and safety; recreation facilities^2^; residential density; crime safety; traffic safety; valid days of wear; average wear time |
| 12D | Direct effects of Having own social media | accessibility and walking facilities; adolescent age; adolescent sex; aesthetics; city; electronic devices in the bedroom; residential density; highest education in household; land use mix - diversity^1^; area-level SES; pedestrian infrastructure and safety; personal electronic devices; recreation facilities^2^; crime safety; traffic safety; valid days of wear; average wear time |
| 13T | Total effects of Electronic devices in the bedroom | accessibility and walking facilities; adolescent age; adolescent sex; aesthetics; city; highest education in household; land use mix - diversity^1^; area-level SES; park proximity; pedestrian infrastructure and safety; recreation facilities^2^; residential density; crime safety; traffic safety; valid days of wear; average wear time |
| 13D | Direct effects of Electronic devices in the bedroom | accessibility and walking facilities; adolescent age; adolescent sex; aesthetics; city; highest education in household; land use mix – diversity^1^; area-level SES; park proximity; pedestrian infrastructure and safety; recreation facilities^2^; residential density; crime safety; traffic safety; transit stop proximity; valid days of wear; average wear time |

*Note.* ^1^ excluding transit stops; ^2^ excluding parks. Potential mediators in *italics.*

**Table S10. Total and direct effects of perceived neighbourhood and home environment characteristics on adolescents’ accelerometer-assessed total sedentary time [complete case analyses; N=3148]**

| **Model** | **Effect estimated** | ***b*** | **95% CI** | ***p*-value** |
| --- | --- | --- | --- | --- |
| 1T | Total effects on Residential density | 0.001 | -0.01, 0.02 | 0.848 |
| 1D | Direct effects of Residential density | -0.0001 | -0.02, 0.02 | 0.994 |
| 2T | Total effects of Land use mix diversity^1^ | -1.21 | -3.83, 1.41 | 0.364 |
| 2D | Direct effects of Land use mix diversity^1^ | 0.22 | -3.15, 3.59 | 0.899 |
| 3T | Total effects of Transit stop proximity | -1.37 | -3.35, 0.60 | 0.172 |
| 3D | Direct effects of Transit stop proximity | -1.29 | -3.51, 0.94 | 0.258 |
| 4T | Total effects of Recreation facilities^2^ | -1.70 | -4.20, 0.79 | 0.181 |
| 4D | Direct effects of Recreation facilities^2^ | -1.44 | -4.72, 1.84 | 0.389 |
| 5T | Total effects of Park proximity | -0.20 | -2.14, 1.74 | 0.840 |
| 5D | Direct effects of Park proximity | 0.91 | -1.49, 3.31 | 0.458 |
| 6T | Total effects of Accessibility and walking facilities | -1.57 | -5.22, 2.08 | 0.399 |
| 6D | Direct effects of Accessibility and walking facilities | -2.60 | -6.66, 1.47 | 0.211 |
| 7T | Total effects of Traffic safety | -1.52 | -4.64, 1.60 | 0.339 |
| 7D | Direct effects of Traffic safety | -0.82 | -4.16, 2.51 | 0.628 |
| 8T | Total effects of Pedestrian infrastructure and safety | -0.92 | -3.93, 2.10 | 0.551 |
| 8D | Direct effects of Pedestrian infrastructure and safety | 0.11 | -3.24, 3.46 | 0.950 |
| 9T | Total effects of Crime safety | -1.00 | -3.38, 1.39 | 0.412 |
| 9D | Direct effects of Crime safety | -0.95 | -3.51, 1.61 | 0.467 |
| 10T | Total effects of Aesthetics | 0.66 | -2.02, 3.34 | 0.628 |
| 10D | Direct effects of Aesthetics | 0.63 | -2.28, 3.53 | 0.673 |
| 11T | Total effects of Personal electronic devices | 1.84 | -0.73, 4.41 | 0.161 |
| 11D | Direct effects of Personal electronic devices | 1.84 | -0.73, 4.41 | 0.161 |
| 12T | Total effects of Having own social media | 4.65 | -0.61, 9.92 | 0.083 |
| 12D | Direct effects of Having own social media | 4.65 | -0.61, 9.92 | 0.083 |
| 13T | Total effects of Electronic devices in the bedroom | 0.25 | -1.07, 1.58 | 0.709 |
| 13D | Direct effects of Electronic thing in the bedroom | 0.25 | -1.07, 1.58 | 0.709 |

*Note.* ^1^ excluding transit stops; ^2^ excluding parks; *b* = regression coefficient; CI = confidence intervals

**Table S11. Child’s sex as a moderator of total and direct effects of perceived neighbourhood and home environment characteristics on adolescents’ accelerometer-assessed total sedentary time**

| Model | Environmental effect estimated | Regression coefficient | ***b*** | **95% CI** | ***p*** |
| --- | --- | --- | --- | --- | --- |
| 1IT* | Total effects of Residential density | Interaction with sex | -0.01 | -0.03, 0.01 | 0.202 |
| 1ID* | Direct effects of Residential density | Interaction with sex | -0.01 | -0.03, 0.01 | 0.378 |
| 2IT* | Total effects of Land use mix diversity^1^ | Interaction with sex | **-6.02** | **-10.48, -1.57** | **0.008** |
|  |  | Males | 2.06 | -1.49, 5.62 | 0.255 |
|  |  | Females | **-3.96** | **-7.26, -0.66** | **0.019** |
| 2ID* | Direct effects of Land use mix diversity^1^ | Interaction with sex | **-5.73** | **-10.23, -1.23** | **0.013** |
|  |  | Males | 3.40 | -0.77, 7.57 | 0.110 |
|  |  | Females | -2.33 | -6.23, 1.57 | 0.241 |
| 3IT* | Total effects of Transit stop proximity | Interaction with sex | **-3.81** | **-7.25, -0.37** | **0.030** |
|  |  | Males | 0.60 | -2.05, 3.25 | 0.657 |
|  |  | Females | **-3.21** | **-5.78, -0.65** | **0.014** |
| 3ID* | Direct effects of Transit stop proximity | Interaction with sex | **-3.83** | **-7.27, -0.39** | **0.029** |
|  |  | Males | 0.76 | -2.05, 3.57 | 0.595 |
|  |  | Females | **-3.07** | **-5.77, -0.37** | **0.026** |
| 4IT* | Total effects of Recreation facilities^2^ | Interaction with sex | **-6.45** | **-11.08, -1.81** | **0.006** |
|  |  | Males | 1.84 | -1.71, 5.40 | 0.310 |
|  |  | Females | **-4.61** | **-7.84, -1.37** | **0.005** |
| 4ID* | Direct effects of Recreation facilities^2^ | Interaction with sex | **-6.32** | **-11.05, -1.58** | **0.009** |
|  |  | Males | 2.05 | -2.12, 6.22 | 0.335 |
|  |  | Females | **-4.26** | **-8.14, -0.39** | **0.031** |
| 5IT* | Total effects of Park proximity | Interaction with sex | **-5.05** | **-8.20, -1.19** | **0.002** |
|  |  | Males | 2.58 | -0.01, 5.17 | 0.051 |
|  |  | Females | **-2.47** | **-4.86, -0.08** | **0.043** |
| 5ID* | Direct effects of Park proximity | Interaction with sex | **-5.14** | **-8.28, -1.99** | **0.001** |
|  |  | Males | **3.11** | **0.38, 5.83** | **0.026** |
|  |  | Females | -2.03 | -4.58, 0.62 | 0.062 |
| 6IT* | Total effects of Accessibility and walking facilities | Interaction with sex | **-12.76** | **-19.36, -6.15** | **<0.001** |
|  |  | Males | **5.24** | **0.19, 10.29** | **0.042** |
|  |  | Females | **-7.52** | **-12.27, -2.77** | **0.002** |
| 6ID* | Direct effects of Accessibility and walking facilities | Interaction with sex | **-13.92** | **-20.66, -7.18** | **<0.001** |
|  |  | Males | 4.88 | -0.53, 10.28 | 0.077 |
|  |  | Females | **-9.05** | **-14.14, -3.96** | **<0.001** |
| 7IT* | Total effects of Traffic safety | Interaction with sex | -3.89 | -9.67, 1.89 | 0.187 |
| 7ID* | Direct effects of Traffic safety | Interaction with sex | -3.89 | -9.67, 1.89 | 0.187 |
| 8IT* | Total effects of Pedestrians infrastructure | Interaction with sex | -1.81 | -7.62, 4.01 | 0.543 |
| 8ID* | Direct effects of Pedestrians infrastructure | Interaction with sex | -1.81 | -7.62, 4.01 | 0.543 |
| 9IT* | Total effects of Crime safety | Interaction with sex | 0.73 | -3.50, 4.96 | 0.735 |
| 9ID* | Direct effects of Crime safety | Interaction with sex | 0.73 | -3.50, 4.96 | 0.735 |
| 10IT* | Total effects of Aesthetics | Interaction with sex | 1.22 | -3.44, 5.88 | 0.609 |
| 10ID* | Direct effects of Aesthetics | Interaction with sex | 1.22 | -3.44, 5.88 | 0.609 |
| 11IT* | Total effects of personal electronic devices | Interaction with sex | -4.18 | -8.57, 0.22 | 0.062 |
| 11ID* | Direct effects of personal electronic devices | Interaction with sex | -4.18 | -8.57, 0.22 | 0.062 |
| 12IT* | Total effects of having own social media | Interaction with sex | 4.73 | -4.78, 14.25 | 0.329 |
| 12ID* | Direct effects of having own social media | Interaction with sex | 4.73 | -4.78, 14.25 | 0.329 |
| 13IT* | Total effects of electronic devices in the bedroom | Interaction with sex | **-2.96** | **-5.48, -0.44** | **0.021** |
|  |  | Males | 1.61 | -0.14, 3.36 | 0.071 |
|  |  | Females | -1.35 | -3.24, 0.54 | 0.162 |
| 13ID* | Direct effects of electronic devices in the bedroom | Interaction with sex | **-2.96** | **-5.48, -0.44** | **0.021** |
|  |  | Males | 1.61 | -0.14, 3.36 | 0.071 |
|  |  | Females | -1.35 | -3.24, 0.54 | 0.162 |

*Note.* ^1^ excluding transit stops; ^2^ excluding parks; *b* = regression coefficient; CI = confidence interval; in bold: effects significant at *p*<0.05

**Table S12. Total and direct effects of perceived neighbourhood and home environment characteristics on adolescents’ accelerometer-assessed sedentary time during out-of-school periods on school days [complete case analyses; N=3148]**

| **Model** | **Effect estimated** | ***b*** | **95% CI** | ***p*-value** |
| --- | --- | --- | --- | --- |
| 1T | Total effects on Residential density | 0.004 | -0.01, 0.01 | 0.401 |
| 1D | Direct effects of Residential density | 0.004 | -0.01, 0.01 | 0.452 |
| 2T | Total effects of Land use mix diversity^1^ | -1.39 | -3.19, 0.40 | 0.128 |
| 2D | Direct effects of Land use mix diversity^1^ | -0.39 | -2.72, 1.93 | 0.740 |
| 3T | Total effects of Transit stop proximity | -0.88 | -2.23, 0.47 | 0.203 |
| 3D | Direct effects of Transit stop proximity | -0.44 | -1.98, 1.09 | 0.572 |
| 4T | Total effects of Recreation facilities^2^ | **-1.86** | **-3.57, -0.15** | **0.033** |
| 4D | Direct effects of Recreation facilities^2^ | -2.10 | -4.37, 0.16 | 0.069 |
| 5T | Total effects of Park proximity | 0.17 | -1.16, 1.51 | 0.797 |
| 5D | Direct effects of Park proximity | **1.69** | **0.04, 3.35** | **0.045** |
| 6T | Total effects of Accessibility and walking facilities | -2.28 | -4.78, 0.22 | 0.074 |
| 6D | Direct effects of Accessibility and walking facilities | -2.20 | -5.01, 0.60 | 0.124 |
| 7T | Total effects of Traffic safety | -1.62 | -3.76, 0.52 | 0.137 |
| 7D | Direct effects of Traffic safety | -1.28 | -3.58, 1.02 | 0.277 |
| 8T | Total effects of Pedestrian infrastructure and safety | **-2.08** | **-4.14, -0.01** | **0.049** |
| 8D | Direct effects of Pedestrian infrastructure and safety | -1.05 | -3.36, 1.27 | 0.375 |
| 9T | Total effects of Crime safety | -0.49 | -2.13, 1.15 | 0.558 |
| 9D | Direct effects of Crime safety | -0.13 | -1.89, 1.64 | 0.882 |
| 10T | Total effects of Aesthetics | 0.13 | -1.71, 1.97 | 0.889 |
| 10D | Direct effects of Aesthetics | 0.85 | -1.16, 2.85 | 0.407 |
| 11T | Total effects of Personal electronic devices | 1.51 | -0.26, 3.27 | 0.095 |
| 11D | Direct effects of Personal electronic devices | 1.51 | -0.26, 3.27 | 0.095 |
| 12T | Total effects of Having own social media | 3.20 | -0.42, 6.81 | 0.830 |
| 12D | Direct effects of Having own social media | 3.20 | -0.42, 6.81 | 0.830 |
| 13T | Total effects of Electronic devices in the bedroom | 0.60 | -0.31, 1.52 | 0.196 |
| 13D | Direct effects of Electronic thing in the bedroom | 0.60 | -0.31, 1.52 | 0.196 |

*Note.*^1^ excluding transit stops; ^2^ excluding parks; *b* = regression coefficient; CI = confidence intervals; in bold: effects significant at *p*<0.05

**Table S13. Child’s sex as a moderator of total and direct effects of perceived neighbourhood and home environment characteristics on adolescents’ accelerometer-assessed to sedentary time during out-of-school periods on school days**

| Model | Environmental effect estimated | Regression coefficient | ***b*** | **95% CI** | ***p*** |
| --- | --- | --- | --- | --- | --- |
| 1IT* | Total effects of Residential density | Interaction with sex | 0.002 | -0.01, 0.02 | 0.748 |
| 1ID* | Direct effects of Residential density | Interaction with sex | 0.003 | -0.01, 0.02 | 0.730 |
| 2IT* | Total effects of Land use mix diversity^1^ | Interaction with sex | -1.85 | -4.90, 1.20 | 0.234 |
| 2ID* | Direct effects of Land use mix diversity^1^ | Interaction with sex | -1.81 | -4.92, 1.30 | 0.255 |
| 3IT* | Total effects of Transit stop proximity | Interaction with sex | **-3.06** | **-5.42, -0.70** | **0.011** |
|  |  | Males | 0.70 | -1.11, 2.52 | 0.447 |
|  |  | Females | **-2.36** | **-4.12, -0.59** | **0.009** |
| 3ID* | Direct effects of Transit stop proximity | Interaction with sex | **-3.40** | **-5.81, -0.99** | **0.006** |
|  |  | Males | 1.34 | -0.64, 3.32 | 0.184 |
|  |  | Females | **-2.06** | **-3.96, -0.15** | **0.034** |
| 4IT* | Total effects of Recreation facilities^2^ | Interaction with sex | **-3.37** | **-6.55, -0.20** | **0.038** |
|  |  | Males | -0.01 | -2.44, 2.43 | 0.966 |
|  |  | Females | **-3.38** | **-5.60, -1.16** | **0.003** |
| 4ID* | Direct effects of Recreation facilities^2^ | Interaction with sex | **-3.82** | **-7.09, 0.55** | **0.022** |
|  |  | Males | 0.02 | -2.87, 2.90 | 0.992 |
|  |  | Females | **-3.80** | **-6.48, -1.13** | **0.005** |
| 5IT* | Total effects of Park proximity | Interaction with sex | **-3.20** | **-5.35, -1.04** | **0.004** |
|  |  | Males | **1.93** | **0.16, 3.71** | **0.033** |
|  |  | Females | -1.26 | -2.90, 0.38 | 0.131 |
| 5ID* | Direct effects of Park proximity | Interaction with sex | **-3.48** | **-5.70, -1.27** | **0.002** |
|  |  | Males | **3.60** | **1.57, 5.64** | **<0.001** |
|  |  | Females | 0.12 | -1.80, 2.04 | 0.902 |
| 6IT* | Total effects of Accessibility and walking facilities | Interaction with sex | **-6.87** | **-11.40, -2.34** | **0.003** |
|  |  | Males | 1.39 | -2.07, 4.85 | 0.432 |
|  |  | Females | **-5.48** | **-8.74, -2.23** | **0.001** |
| 6ID* | Direct effects of Accessibility and walking facilities | Interaction with sex | **-7.89** | **-12.55, -3.32** | **0.001** |
|  |  | Males | 2.02 | -1.71, 5.76 | 0.288 |
|  |  | Females | **-5.87** | **-9.38, -2.35** | **0.001** |
| 7IT* | Total effects of Traffic safety | Interaction with sex | -2.18 | -6.14, 1.78 | 0.280 |
| 7ID* | Direct effects of Traffic safety | Interaction with sex | -2.46 | -6.51, 1.60 | 0.235 |
| 8IT* | Total effects of Pedestrian infrastructure | Interaction with sex | -0.79 | -4.78, 3.19 | 0.696 |
| 8ID* | Direct effects of Pedestrian infrastructure | Interaction with sex | -1.97 | -6.08, 2.13 | 0.346 |
| 9IT* | Total effects of Crime safety | Interaction with sex | -0.99 | -3.89, 1.92 | 0.506 |
| 9ID* | Direct effects of Crime safety | Interaction with sex | -1.76 | -4.78, 1.25 | 0.252 |
| 10IT* | Total effects of Aesthetics | Interaction with sex | -0.76 | -3.96, 2.45 | 0.644 |
| 10ID* | Direct effects of Aesthetics | Interaction with sex | -1.28 | -4.64, 2.07 | 0.454 |
| 11IT* | Total effects of personal electronic devices | Interaction with sex | **-3.11** | **-6.12, -0.10** | **0.043** |
|  |  | Males | **3.04** | **0.74, 5.33** | **0.009** |
|  |  | Females | -0.07 | -2.40, 2.25 | 0.950 |
| 11ID* | Direct effects of personal electronic devices | Interaction with sex | **-3.11** | **-6.12, -0.10** | **0.043** |
|  |  | Males | **3.04** | **0.74, 5.33** | **0.009** |
|  |  | Females | -0.07 | -2.40, 2.25 | 0.950 |
| 12IT* | Total effects of having own social media | Interaction with sex | 2.70 | -3.83, 9.23 | 0.418 |
| 12ID* | Direct effects of having own social media | Interaction with sex | 2.70 | -3.83, 9.23 | 0.418 |
| 13IT* | Total effects of electronic devices in the bedroom | Interaction with sex | **-2.44** | **-4.18, -0.70** | **0.006** |
|  |  | Males | **1.72** | **0.51, 2.93** | **0.005** |
|  |  | Females | -0.72 | -2.02, 0.59 | 0.281 |
| 13ID* | Direct effects of electronic devices in the bedroom | Interaction with sex | **-2.44** | **-4.18, -0.70** | **0.006** |
|  |  | Males | **1.72** | **0.51, 2.93** | **0.005** |
|  |  | Females | -0.72 | -2.02, 0.59 | 0.281 |

*Note.* ^1^ excluding transit stops; ^2^ excluding parks; *b* = regression coefficient; CI = confidence interval; in bold: effects significant at *p*<0.05

**Table S14. Total and direct effects of perceived neighbourhood and home environment characteristics on adolescents’ accelerometer-assessed sedentary time on non-school days [complete case analyses; N=3148]**

| **Model** | **Effect estimated** | ***b*** | **95% CI** | ***p*-value** |
| --- | --- | --- | --- | --- |
| 1T | Total effects on Residential density | 0.01 | -0.01, 0.03 | 0.283 |
| 1D | Direct effects of Residential density | 0.01 | -0.01, 0.03 | 0.482 |
| 2T | Total effects of Land use mix diversity^1^ | 2.50 | -1.00, 6.01 | 0.162 |
| 2D | Direct effects of Land use mix diversity^1^ | 3.89 | -0.60, 8.39 | 0.089 |
| 3T | Total effects of Transit stop proximity | -1.51 | -4.15, 1.13 | 0.261 |
| 3D | Direct effects of Transit stop proximity | **-3.19** | **-6.18, -0.20** | **0.037** |
| 4T | Total effects of Recreation facilities^2^ | 0.94 | -2.39, 4.28 | 0.579 |
| 4D | Direct effects of Recreation facilities^2^ | 1.56 | -2.82, 5.94 | 0.486 |
| 5T | Total effects of Park proximity | -0.41 | -3.02, 2.19 | 0.756 |
| 5D | Direct effects of Park proximity | -1.65 | -4.89, 1.58 | 0.316 |
| 6T | Total effects of Accessibility and walking facilities | 1.52 | -3.35, 6.38 | 0.541 |
| 6D | Direct effects of Accessibility and walking facilities | 0.76 | -4.65, 6.18 | 0.783 |
| 7T | Total effects of Traffic safety | -0.21 | -4.36, 3.94 | 0.921 |
| 7D | Direct effects of Traffic safety | 0.31 | -4.13, 4.75 | 0.891 |
| 8T | Total effects of Pedestrians infrastructure and safety | -0.37 | -4.39, 3.65 | 0.856 |
| 8D | Direct effects of Pedestrians infrastructure and safety | -0.38 | -4.09, 4.85 | 0.868 |
| 9T | Total effects of Crime safety | -0.91 | -4.11, 2.28 | 0.574 |
| 9D | Direct effects of Crime safety | -0.23 | -3.67, 3.20 | 0.895 |
| 10T | Total effects of Aesthetics | -0.94 | -4.51, 2.62 | 0.605 |
| 10D | Direct effects of Aesthetics | -2.27 | -6.14, 1.59 | 0.249 |
| 11T | Total effects of Personal electronic devices | 2.85 | -0.57, 6.26 | 0.103 |
| 11D | Direct effects of Personal electronic devices | 2.85 | -0.57, 6.26 | 0.103 |
| 12T | Total effects of Having own social media | 4.88 | -2.12, 11.88 | 0.172 |
| 12D | Direct effects of Having own social media | 4.88 | -2.12, 11.88 | 0.172 |
| 13T | Total effects of Electronic devices in the bedroom | 1.49 | -0.28, 3.26 | 0.099 |
| 13D | Direct effects of Electronic thing in the bedroom | 1.49 | -0.28, 3.26 | 0.099 |

*Note.* ^1^ excluding transit stops; ^2^ excluding parks; *b* = regression coefficient; CI = confidence intervals; in bold: effects significant at *p*<0.05

**Table S15. Child’s sex as a moderator of total and direct effects of perceived neighbourhood and home environment characteristics on adolescents’ accelerometer-assessed to sedentary time on non-school days**

| Model | Environmental effect estimated | Regression coefficient | ***b*** | **95% CI** | ***p*** |
| --- | --- | --- | --- | --- | --- |
| 1IT* | Total effects of Residential density | Interaction with sex | -0.01 | -0.04, 0.02 | 0.455 |
| 1ID* | Direct effects of Residential density | Interaction with sex | -0.01 | -0.03, 0,02 | 0.648 |
| 2IT* | Total effects of Land use mix diversity^1^ | Interaction with sex | **-6.50** | **-12.45, -0.54** | **0.032** |
|  |  | Males | **5.99** | **1.70, 10.71** | **0.013** |
|  |  | Females | 1.02 | -4.17, 6.22 | 0.700 |
| 2ID* | Direct effects of Land use mix diversity^1^ | Interaction with sex | **-6.45** | **-12.47, -0.42** | **0.036** |
|  |  | Males | **7.47** | **1.91, 13.04** | **0.009** |
|  |  | Females | 1.02 | -4.17, 6.22 | 0.700 |
| 3IT* | Total effects of Transit stop proximity | Interaction with sex | **-4.51** | **-9.11, 0.09** | **0.055** |
|  |  | Males | 0.79 | -2.73, 4.31 | 0.660 |
|  |  | Females | **-3.72** | **-7.17, -0.27** | **0.035** |
| 3ID* | Direct effects of Transit stop proximity | Interaction with sex | **-4.71** | **-9.38, -0.04** | **0.048** |
|  |  | Males | -0.75 | -4.57, 3.07 | 0.700 |
|  |  | Females | **-5.46** | **-9.18, -1.75** | **0.004** |
| 4IT* | Total effects of Recreation facilities^2^ | Interaction with sex | **-6.43** | **-12.63, -0.22** | **0.042** |
|  |  | Males | 4.43 | -0.29, 9.16 | 0.066 |
|  |  | Females | -2.00 | -6.36, 2.36 | 0.369 |
| 4ID* | Direct effects of Recreation facilities^2^ | Interaction with sex | **-7.11** | **-13.46, -0.77** | **0.028** |
|  |  | Males | 5.46 | -0.10, 11.02 | 0.054 |
|  |  | Females | -1.65 | -6.85, 3.55 | 0.534 |
| 5IT* | Total effects of Park proximity | Interaction with sex | **-6.80** | **-11.03, -2.57** | **0.002** |
|  |  | Males | 3.28 | -0.18, 6.74 | 0.063 |
|  |  | Females | **-3.52** | **-6.75, -0.29** | **0.033** |
| 5ID* | Direct effects of Park proximity | Interaction with sex | **-6.82** | **-11.12, -2.51** | **0.002** |
|  |  | Males | 2.03 | -1.93, 5.99 | 0.316 |
|  |  | Females | **-4.79** | **-8.56, -1.02** | **0.013** |
| 6IT* | Total effects of Accessibility and walking facilities | Interaction with sex | **-17.81** | **-26.61, -9.01** | **<0.001** |
|  |  | Males | **10.90** | **4.22, 17.59** | **0.001** |
|  |  | Females | **-6.91** | **-13.28, -0.53** | **0.034** |
| 6ID* | Direct effects of Accessibility and walking facilities | Interaction with sex | **-18.90** | **-27.88, -9.91** | **<0.001** |
|  |  | Males | **10.79** | **3.63, 17.95** | **0.003** |
|  |  | Females | **-8.11** | **-14.92, -1.30** | **0.020** |
| 7IT* | Total effects of Traffic safety | Interaction with sex | -2.89 | -10.59, 4.80 | 0.461 |
| 7ID* | Direct effects of Traffic safety | Interaction with sex | -2.70 | -10.51, 5.12 | 0.499 |
| 8IT* | Total effects of Pedestrian infrastructure | Interaction with sex | -0.04 | -7.83, 7.75 | 0.992 |
| 8ID* | Direct effects of Pedestrian infrastructure | Interaction with sex | -1.63 | -9.60, 6.34 | 0.689 |
| 9IT* | Total effects of Crime safety | Interaction with sex | 1.61 | -4.05, 7.28 | 0.576 |
| 9ID* | Direct effects of Crime safety | Interaction with sex | 0.95 | -4.91, 6.80 | 0.751 |
| 10IT* | Total effects of Aesthetics | Interaction with sex | 3.26 | -2.98, 9.49 | 0.306 |
| 10ID* | Direct effects of Aesthetics | Interaction with sex | 1.92 | -4.58, 8.43 | 0.563 |
| 11IT* | Total effects of personal electronic devices | Interaction with sex | -3.25 | -9.09, 2.59 | 0.275 |
| 11ID* | Direct effects of personal electronic devices | Interaction with sex | -3.25 | -9.09, 2.59 | 0.275 |
| 12IT* | Total effects of having own social media | Interaction with sex | 4.83 | -7.81, 17.47 | 0.454 |
| 12ID* | Direct effects of having own social media | Interaction with sex | 4.83 | -7.81, 17.47 | 0.454 |
| 13IT* | Total effects of electronic devices in the bedroom | Interaction with sex | -2.76 | -6.13, 0.62 | 0.109 |
| 13ID* | Direct effects of electronic devices in the bedroom | Interaction with sex | -2.76 | -6.13, 0.62 | 0.109 |

*Note.* ^1^ excluding transit stops; ^2^ excluding parks; *b* = regression coefficient; CI = confidence interval; in bold: effects significant at *p*<0.05

**Summary of findings:**

No significant total or direct main effects of perceived neighbourhood and home environment characteristics with accelerometer-assessed total sedentary time were observed, although adolescents their own social media tended to accumulate more sedentary time (Table S10). In contrast, parent-perceived access to recreation facilities and pedestrian infrastructure and safety showed significant negative associations with accelerometer-assessed sedentary time during out-of-school periods on school days in the total-effect models, and park proximity showed a positive association in the direct-effect models (Table S12). A weak, albeit non-significant, positive association was also found between having personal electronic devices at home and the same sedentary time outcome. A significant negative direct effect was observed between transit stop proximity and accelerometer-assessed sedentary time on non-school days, while marginal positive associations were found in relation to land use mix – diversity (direct effect) and electronic devices in the bedroom (Table S14).

Adolescents’ sex moderated a substantial number of associations between perceived environmental characteristics and accelerometer-assessed sedentary time outcomes. Only females displayed negative associations of accelerometer-assessed total sedentary time with land use mix – diversity, transit stop proximity, access to recreation facilities, park proximity and accessibility and walking facilities (Table S11). In contrast, the associations of total sedentary time with park proximity and accessibility and walking facilities tended to be positive. The same was observed for electronic devices in the bedroom. Several of these sex-specific associations held for accelerometer-assessed sedentary time during out-of-school periods on school days (Table S13) and on non-school days (Table S15). Sedentary time during out-of-school periods on school days was negatively related to transit stop proximity, recreation facilities and accessibility and walking facilities only in females, and positively related to park proximity and electronic devices in the bedroom only in males. In addition, males, but not females, with more personal electronic devices tended to accumulate more sedentary time during out-of-school periods on school days (Table S13). In females, transit stop proximity, park proximity and accessibility and walking facilities were also negatively related to sedentary time on non-school days (Table S15). In contrast, males displayed positive relations of land use mix – diversity and accessibility and walking facilities with sedentary time on non-school days.

No significant between-city differences were found in the associations between perceived environmental characteristics and accelerometer-assessed sedentary time outcomes.
